# Progress in the pharmacological treatment of human cystic and alveolar echinococcosis: Compounds and therapeutic targets

**DOI:** 10.1371/journal.pntd.0006422

**Published:** 2018-04-20

**Authors:** Mar Siles-Lucas, Adriano Casulli, Roberto Cirilli, David Carmena

**Affiliations:** 1 Group of Parasitology, IRNASA-CSIC, Salamanca, Spain; 2 World Health Organization Collaborating Centre for the Epidemiology, Detection and Control of Cystic and Alveolar Echinococcosis, Istituto Superiore di Sanità, Rome, Italy; 3 European Union Reference Laboratory for Parasites (EURLP), Istituto Superiore di Sanità, Rome, Italy; 4 National Centre for the Control and Evaluation of Medicines, Istituto Superiore di Sanità, Rome, Italy; 5 Parasitology Reference and Research Laboratory, National Centre for Microbiology, Carlos III Health Institute, Majadahonda, Madrid, Spain; Gesundheitsförderung Schweiz, SWITZERLAND

## Abstract

Human cystic and alveolar echinococcosis are helmintic zoonotic diseases caused by infections with the larval stages of the cestode parasites *Echinococcus granulosus* and *E*. *multilocularis*, respectively. Both diseases are progressive and chronic, and often fatal if left unattended for *E*. *multilocularis*. As a treatment approach, chemotherapy against these orphan and neglected diseases has been available for more than 40 years. However, drug options were limited to the benzimidazoles albendazole and mebendazole, the only chemical compounds currently licensed for treatment in humans. To compensate this therapeutic shortfall, new treatment alternatives are urgently needed, including the identification, development, and assessment of novel compound classes and drug targets. Here is presented a thorough overview of the range of compounds that have been tested against *E*. *granulosus* and *E*. *multilocularis* in recent years, including *in vitro* and *in vivo* data on their mode of action, dosage, administration regimen, therapeutic outcomes, and associated clinical symptoms. Drugs covered included albendazole, mebendazole, and other members of the benzimidazole family and their derivatives, including improved formulations and combined therapies with other biocidal agents. Chemically synthetized molecules previously known to be effective against other infectious and non-infectious conditions such as anti-virals, antibiotics, anti-parasites, anti-mycotics, and anti-neoplastics are addressed. In view of their increasing relevance, natural occurring compounds derived from plant and fungal extracts are also discussed. Special attention has been paid to the recent application of genomic science on drug discovery and clinical medicine, particularly through the identification of small inhibitor molecules tackling key metabolic enzymes or signalling pathways.

## Author summary

Human cystic and alveolar echinococcosis (CE and AE), caused by the larval stages of the helminths *Echinococcus granulosus* and *E*. *multilocularis*, respectively, are progressive and chronic diseases affecting more than 1 million people worldwide. Both are considered orphan and neglected diseases by the World Health Organization. As a treatment approach, chemotherapy is limited to the use of benzimidazoles, drugs that stop parasite growth but do not kill the parasite. To compensate this therapeutic shortfall, new treatment alternatives are urgently needed. Here, we present the state-of-the-art regarding the alternative compounds and new formulations of benzimidazoles assayed against these diseases until now. Some of these new and modified compounds, either alone or in combination, could represent a step forward in the treatment of CE and AE. Unfortunately, few compounds have reached clinical trials stage in humans and, when assayed, the design of these studies has not allowed evidence-based conclusions. Thus, there is still an urgent need for defining new compounds or improved formulations of those already assayed, and also for a careful design of clinical protocols that could lead to the draw of a broad international consensus on the use of a defined drug, or a combination of drugs, for the effective treatment of CE and AE.

## Introduction

Human cystic and alveolar echinococcosis (CE and AE) are chronic diseases caused by the larval stages of the cestodes *Echinococcus granulosus* sensu lato (s.l.) and *E*. *multilocularis*, respectively. Both parasites can develop in humans after ingestion of the infective eggs of the parasite shed in the faeces of canid definitive hosts. The oncospheres released from the eggs in the intestine penetrate the gut wall and enter the blood vessels, reaching different organs, mainly liver and lungs, in which they develop into the asexual cystic stage. In AE proliferative metacestode vesicles intermingle with host connective tissue and immune cells and grow steadily and in an infiltrative manner resembling that of cancer progression, while CE single or multiple cyst growth is slow and the living parasite tissue remains enclosed by the external laminated and adventitial layers, resulting in a fluid-filled spherical shaped lesion [1,2].

Treatment options for AE are few, with surgery reserved for early stages of the infection when lesions can be completely resected and there are not distant metastases, followed by prophylactic anti-parasite drug treatment. Savage treatment may be attempted in advanced cases by major palliative surgery or even liver transplantation, whereas in inoperable cases treatment relies on prolonged administration of benzimidazole drugs. For CE four treatment options have been suggested based on the WHO classification of cyst stages as seen in imaging-based techniques: percutaneous interventions [Percutaneous-Aspiration-Injection and Re-Aspiration (PAIR) and its modifications, percutaneous evacuation (PEVAC), Modified Catheterization Technique (MoCaT), and Dilatable Multi-Function Trocar (DMFT)], surgery, chemotherapy, and a watch-and-wait approach. For the first two options, pre- and post-treatment and peri-interventional treatment with anti-parasitic drugs is also used [3].

As a rule of thumb, the ideal agent for the chemotherapeutic treatment of CE and AE should comply with as many as possible of the following features: it should be selectively toxic against the parasite (but not the host), and endowed with a favorable pharmacokinetics enabling good solubility, absorption, and stability to reach the target site at a concentration sufficient to trigger an effective response at the tissue and intra-cystic levels. A parasitocidal, rather than a parasitostatic effect is also highly desirable, together with a lack of undesirable clinical effects.

Currently available drugs against CE and AE in clinical settings are mainly limited to benzimidazoles (BMZ), and more specifically to albendazole and alternatively mebendazole when albendazole is not well tolerated. BMZ, either alone or in combination with other adjuvantive scolicidal compounds, are also used to prevent recurrence due to protoscoleces spillage following surgery or percutaneous treatment in CE [4].

Although efficacy of BMZ has been proved against CE and AE, this relies in several variables. In CE, efficacy of BMZ varies depending on number, size, type, and localization of cysts that probably influence the penetration of drugs, thus the bioavailability of the compound inside the cyst, and on the age of the patient [5], while the optimal dosage and duration of treatment have never been formally assessed in randomized clinical trials. For AE, long-term BMZ therapy improves the survival rate in non-radically operated patients and prevent recurrences after radical surgery compared to untreated patients [3]. Nevertheless, several studies support the idea that BMZ exert a parasitostatic rather than a parasitocidal effect, particularly against *E*. *multilocularis* lesions, and thus recurrence rates are frequently reported after treatment interruption [5,6].

Alternative drugs and several natural compounds previously known to be effective against different infectious and non-infectious diseases have been also tested in *in vitro* and *in vivo* models of the *Echinococcus* species complex, but only few have reached clinical use [7]. None of them have been specifically designed for the treatment of CE and AE (e.g. the protein kinase inhibitors experimentally assessed against CE or AE have been previously used in cancer treatment as targeted therapy), mainly because the development of orphan drugs for these neglected diseases is of very limited interest to the pharmaceutical industry. Similarly, a group of 400 representative compounds active against malaria, called the “Malaria box”, have also been tested against a variety of disease pathogens [8]. Preliminary studies have demonstrated tha some Malaria box compounds have consistent activity against helminths including *Brugia malayi*, *Trichuris trichiura*, *Ancylostoma ceylanicum*, and *Echinococcus* [8,9], and may represent candidate molecules to advance *Echinococcus* drug development research.

In the following sections we summarize the methods used to assess drug efficacy against CE and AE. We also present an overview of the different compounds that have been tested against *E*. *granulosus* and *E*. *multilocularis* protoscoleces and cysts/vesicles, including data on their mode of action when available, dosage, and therapeutic outcomes.

### Methods to assess drug effects and efficacy against CE and AE

In human infections, established *E*. *granulosus* cysts (or metacestodes) can develop and reach the mature, fertile state. Protoscoleces are then produced from the germinative layer inside the cyst. Spillage of viable protoscoleces after spontaneous or traumatic cyst rupture or during surgical intervention can give rise to new cysts (recurrence). Drugs against CE have been tested both against the metacestode and against the protoscoleces. In AE patients the metacestode usually does not produce protoscoleces, and the majority of the studies carried out to assess the therapeutic efficacy of drugs against AE have been done in the larval stage of the parasite. However, some authors have also tested defined drugs against *E*. *multilocularis* protoscoleces isolated from *in vivo* metacestodes obtained in the murine model [10]. For both CE and AE, activity of the compounds can be assayed *in vitro* and *in vivo*, although the number of agents reaching human clinical trial has been very limited. Lately, drugs have also been tested *in vitro* against stem cells derived from the germinative layer of the metacestode [11,12], both for CE and AE. Measurement of drug activity against protoscoleces is mainly directed towards the identification of effective compounds to reduce the risk of CE recurrence after surgery. The parasitocidal effect of drugs against protoscoleces can be measured *in vitro* using simple procedures involving vital staining with eosin or other vital dyes [13]. Some authors combine this vital staining with the investigation of the ultra-structural changes originated after drug exposure as seen in electron microscopy [14], the measurement of indirect markers of parasite damage including nucleosomal fragmentation and apoptosis-related enzyme activities in treated protoscoleces [14], and, in few cases, the assessment of cyst formation capacity of *in vitro* treated protoscoleces after intraperitoneal injection into rodents, compared with non-treated parasites [15]. A novel movement-based assay has been recently developed for *in vitro*-drug screening using *E*. *multilocularis* protoscoleces cultured in microwell plates. Morphological effects caused by the active compounds tested are then directly measured and quantified by image analysis [10]. Assays against protoscoleces can also be done after the intra-cyst inoculation of the drug to test its scolicidal activity [16], and by the administration of the drug to rodent models shortly before or after intraperitoneal infection with viable protoscoleces to mimic accidental spillage in the peritoneal cavity during a surgical intervention [17].

In general, drug testing against protoscoleces in any of those modalities is of advantage to translate the results mainly to avoid secondary CE in patients. As we mention hereinafter, a number of drugs and compounds have shown good protoscolicidal activity. Thus, drugs against the metacestode are much more urgently needed.

**Fig 1:**
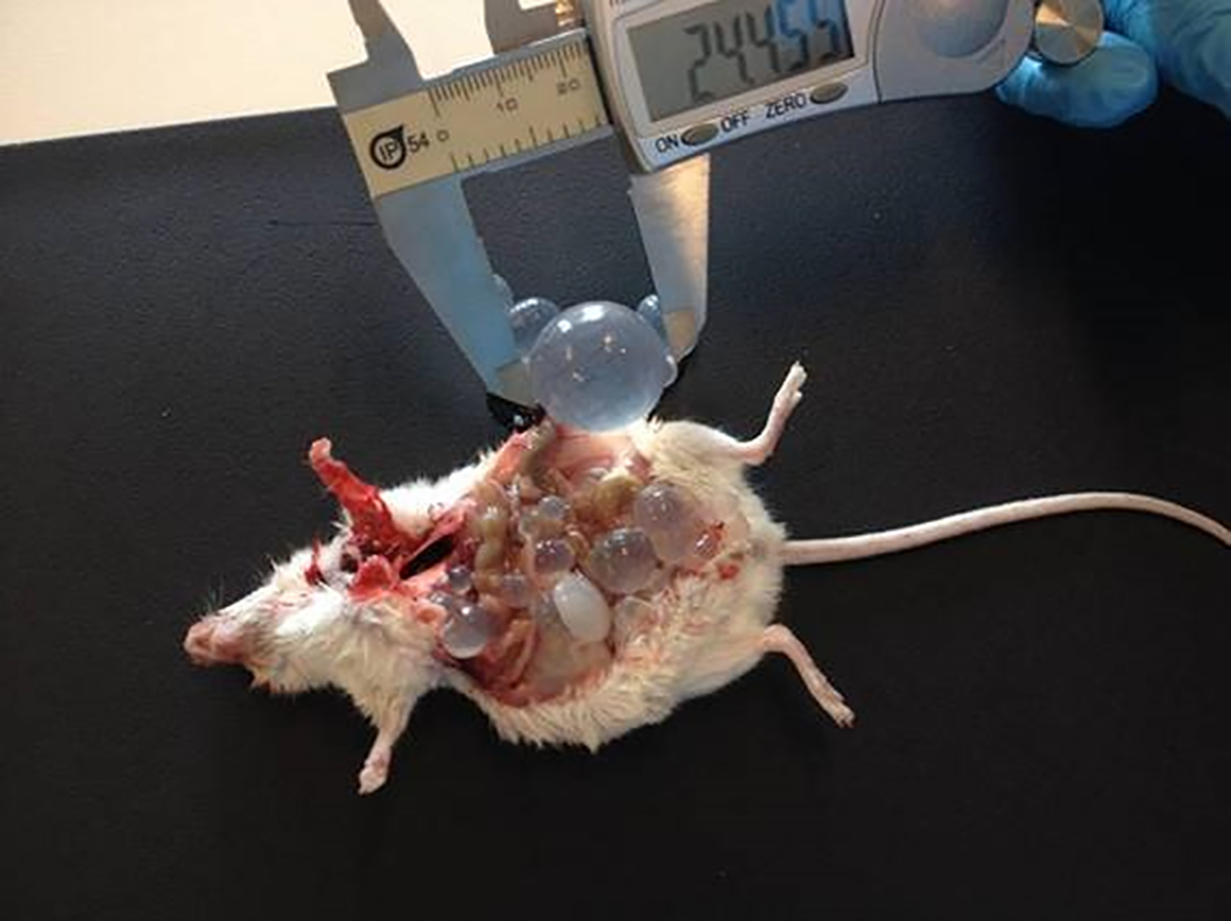
Mouse experimentally infected with *Echinococcus granulosus* sensu stricto. Cysts of *E*. *granulosus* in the peritoneal cavity of an infected mouse. Image credit: Dr. Gulay Vural, Namik Kemal University, Turkey.

Drug activity can be measured *in vitro* against both *E*. *granulosus* s.l. and *E*. *multilocularis* metacestodes maintained in culture, although *E*. *multilocularis* is the preferred experimental model due to the relative simplicity in obtaining parasite material from experimentally infected mice and the feasibility of maintaining and multiplying *in vitro* cultured vesicles of the parasite compared to *E*. *granulosus* cysts (Fig 1) [5,18]. However, the *in vitro* vesicular model has some limitations regarding the extrapolation of the *in vitro* effects to the *in vivo* scenario. First, assessment of total loss of parasite viability after treatment (parasitocidal effect) is difficult. This has been most frequently approached by the study of macroscopic alterations (e.g., shrinking of cysts or detachment of the germinative layer) [19], microscopical changes in the germinative layer (destruction of the cells in the layer) [20], or leaking of parasite-derived compounds to the culture medium and subsequent assessment of their associated enzyme (e.g. alkaline phosphatase, phosphoglucoisomerase) activity [5,21,22]. Although these assays correlate well with parasite damage, parasitocidal activity could only be totally proven after inoculation of the treated parasitic material into a rodent model, or, alternatively, re-culturing the material *in vitro* in order to prove the lack of parasite growth. Second, the development of the cysts in primary infections in natural hosts (after oral infection with parasite eggs) differs from the development of the cysts *in vitro*, both for CE and AE. For CE, cyst growth usually leads to the formation of the external laminated and adventitial layers of variable thickness [23]. In AE patients, cysts grow forming a stromatous mass [23]. In both cases, access of drugs to the living parasite cells is more limited than in the *in vitro* conditions. In addition, because cells growing *in vitro* are not the exact dissociated replicates of their *in vivo* counterparts, *in vitro* models do not take into consideration the potentially synergistic antiparasitic effect of the host's immune system, which may, at least partially, explain some of the outcomes observed in *in vivo* models. Nevertheless, the *in vitro* vesicular model has shown to be very valuable for the high-throughput screening of compounds, specially applying defined assays that show to correlate well with the loss of parasite activity, e.g. the measurement of phosphoglucoisomerase (PGI) activity in the culture medium after drug exposure, avoiding the unnecessary use of experimental animals [24]. Nevertheless, concentration range of drugs tested *in vitro* should consider that bioavilability *in vivo*, both in plasma and intra-cyst, can be three to ten-fold lower than in the *in vitro* systems (e.g., see “Albendazole” section). Additionally, toxicological and adverse effects should be taken into account when high-dose *in vitro* studies are to be translated to *in vivo* conditions.

Similar limitations, although less pronounced, are found in the *in vivo* rodent models. These are usually based on the experimentally-induced development of the parasite after intraperitoneal injection of viable protoscoleces (for *E*. *granulosus*) or micro-cyst (for *E*. *multilocularis*) into rodents (secondary infection) [5]. Development of the parasite within the peritoneal cavity resembles more accurately the situation of a recurrence in CE natural hosts than that of a primary infection. However, this approach has some limitations when attempting to recreate a natural (usually primary liver) infection in the natural host of *E*. *multilocularis*. It should also be noted that intraperitoneal rodent models may be hampered by variations in the efficacy of systemic drug treatments. Differences in cyst location (e.g., intraperitoneal *vs*. intraparenchymatous) may have important consequences in terms of parasite's exposure and achievable tissue concentration of the drug. Additionally, the elected time point after infection to start the drug treatment is of importance for the outcome of the treatment [25]. In few cases, the treatment has been done directly in naturally infected animals for CE, such as sheep [26]. The *in vivo* models have been also used to assess parasite viability after *in vitro* treatment [27]. Assessment of parasite damage after treatment in *in vivo* models has been usually done by estimating the mean cyst number and, more frequently, the mean biomass weight of cysts developed in infected, treated rodents compared with those figures from infected, non-treated animals [13]. Occasionally, drug-induced cyst damage and scolicidal activity has been assessed macro- and microscopically in *in vivo* models [28]. When only cyst weight differences are found, cysticidal activity cannot be guaranteed and instead a parasitostatic effect could be attributed to the tested drug.

Of special importance is the assessment of drug effectiveness against the stem cells of the parasite in AE, since the uncontrolled growth of the metacestode and the growing foci distant from the primary lesion are presumably propitiated by the stem cells present in the germinative layer of *E*. *multilocularis* [29]. The recent availability of the genome and transcriptome data for both *E*. *granulosus* and *E*. *multilocularis* is also of importance for the definition of new drugs that could target defined parasite-specific molecules [11,30,31].

Very few drugs have reached trials in patients. The best characterized drugs are BMZ, which have been use both against CE and AE for many years. Lately, recommendations on the use of BMZ for the stage-specific treatment of CE and AE patients have been produced [3]. Nevertheless, evidence-based data supporting specific dosage and duration of BMZ treatment, either for the prevention of recurrence in CE due to protoscoleces spillage or for the fully effective elimination of the parasite, are not available to date. For other drugs different from BMZ, assessment of treatment efficacy in patients has been usually done retrospectively (e.g., for praziquantel) [32], and in a low number of patients with heterogeneous clinical pictures and management, precluding the extraction of sound conclusions and hampering the generalization of the obtained results [3].

### Benzimidazoles

#### General features

Initially proposed as anthelmintic agents against gastrointestinal parasites of domestic animals in 1961 [33], benzimidazole-carbamate compounds (BMZ) are heterocyclic aromatic organic molecules now widely used in human and veterinary medicine. Members of this large chemical family have demonstrated an extensive therapeutic activity as anti-parasitic (including anthelmintic), anti-bacterial, anti-cancer, anti-fungal, anti-inflammatory, anti-HIV, anti-oxidant, anti-tubercular, and anti-diabetic agents [34]. The generic mechanism of action of BMZ is based on the interference with the assembly and disassembly of the cytoskeletal protein tubulin into microtubule polymers and subsequent inhibition of microtubule-mediated cellular processes, including cell division. BMZ also interfere with mechanisms of glucose uptake by the larval stage of the parasite, leading to glycogen depletion and subsequent degenerative changes in the mitochondria and the endoplasmic reticulum [35,36]. Additionally, an early study has reported mebendazole as inhibitor of the mitochondrial fumarate reductase [37], an enzyme involved in the anaerobic respiration metabolism of helminthes that has been recently postulated as a potential target for chemotherapy [38].

BMZ are extensively metabolized in mammals following administration. Typically, the parent compound is short lived and has poor water solubility, a feature with important implications in the rate and extent of absorption and associated therapeutic efficacy. The primary metabolites, which are generally more soluble than the parent compound, are usually results from oxidative and hydrolytic processes and may be found in plasma, tissue and excreta. Detection of metabolites in urine and faeces may be indicative of poor bioavailability, which is highly influenced by a number of factors including formulation, solubility, and dosage used.

Among BMZ derivatives, albendazole and mebendazole are the only drugs currently licensed for the treatment of human CE and AE [3,39]. Importantly, a large meta-analysis study based on case series from six research centres in five countries comprising 711 treated CE patients have cast doubts on overoptimistic expectations of the overall efficacy of BMZ [40]. Thus, although BMZ therapy was particularly suited for the treatment of small, active cysts (diameter < 6 cm), multi-vesicular, multi-septated, and transitional cysts responded poorly to BMZ and were associated to a high rate of relapses [40]. Other factors known to influence the outcome of the treatment were the age of the patient (BMZ were more effective in younger than in older patients), and the organ affected (e.g. bone cysts were less susceptible to BMZ than hepatic or pulmonary cysts) [5]. Therefore, it is now widely accepted that chemotherapy with albendazole and mebendazole exhibits a parasitostatic rather than a parasitocidal effect, as demonstrated by the elevated recurrent rates frequently observed after interruption of therapy [7]. Although these drugs are generally well tolerated, their target is the parasite tubulin, a conserved molecule very similar in humans, whose undesirable targeting is most probably the cause of adverse reactions. Hepatotoxicity, alopecia, gastrointestinal disturbances, thrombocytopenia, and severe leukopenia have been reported and may require stopping the prescribed treatment [4]. Albendazole and mebendazole may also induce embryotoxic or teratogenic effects [41]. Taking together these facts have prompted the search of new compounds and formulations with enhanced activity against both CE and AE [7,42,43]. Therefore, a number of other BMZ analogs including fenbendazole, flubendazole, oxfendazole, and triclabendazole have been evaluated in recent years both in *in vitro* studies and *in vivo* experimental animal models with different results (see below). Less frequently, studies to increase BMZ bioavailability while avoding a rise in adverse effects have been done (see below).

Relevant *in vitro* and *in vivo* data concerning the chemotherapeutic efficacy of BMZ and their derivatives for the treatment of CE and AE reported in the literature from 2008 to date have been comprehensively reviewed and summarized in Table 1. For information published before this period, the interested reader is referred to a number of excellent review articles in the subject [4–6,42–45].

**Table 1:** Characteristics of benzimidazole compounds, including their metabolites and solubility-improved formulations, as monotherapy for the treatment of cystic and alveolar echinococcosis based on the published literature from 2008 to date.

Compound	Disease	Assay setting			Dosage	Treatment		Efficacy assessment			Reference
		*In vitro*	*In vivo*	In patients (*n*)		Initiation (p.i.)	Duration	Activity against	Success^a^ (%)	Toxicity	
Albendazole	CE	Yes	Yes^b^	—	10 nmol·mL^-1^ (*in vitro*)	N/A (*in vitro*)	30 days (*in vitro*)	PSC	Yes (77, *in vitro*)	N/S	[13]
					5 mg·kg^-1^ (*in vivo*)^e^	6 months(*in vivo*)	25 days (*in vivo*)		No (14, *in vivo*)		
		—	Yes^b^	—	50 mg·kg^-1,^^e^	4 months	45 days	Cysts	Yes (14)	N/S	[28]
		—	Yes^b^	—	150 mg·kg^-1^·day^-1,^^i^	At infection	10 days	Cysts	Yes (100)	N/S	[88]
		—	Yes^b^	—	300 mg·kg^-1^·day^-1,^^i^	8 months	20 days	Cysts	Yes (98)	N/S	[88]
		—	Yes^b^	—	300 mg·kg^-1^·day^-1,^^i^	8 months	20 days	Cysts	Yes (95)	N/S	[89]
		—	Yes^b^	—	25 mg·kg^-1,^^e^	At infection	30 days	Cysts	Yes (89)	N/S	[27]
		—	Yes^b^	—	25 mg·kg^-1,^^e^	4 months	30 days	Cysts	Yes (98)	N/S	[27]
		—	Yes^c^	—	2%^g^	N/A	Single dose	Cysts	Yes (100)	N/S	[90]
		—	Yes^d^	—	8.5 mg·kg^-1,^^e^	N/A	55 days	Cysts	Yes (97)	N/S	[91]
		—	—	Yes (1)	800 mg·day^-1,^^e^	Peri-operative	15 months	None	No (—)	N/S	[156]
		—	—	Yes (27)	10–15 mg·kg^-1^·day^-1,^^e^	Variable	3 months	Cysts	Yes (26–34)	None	[80]
		—	—	Yes (1)	15 mg·kg^-1^·day^-1,^^e^	At diagnosis	5 months	Cysts	Yes (100)	N/S	[217]
		—	—	Yes (26)	10 mg·kg^-1^·day^-1,^^e^	Peri-operative	1 week	Cysts	Yes (100)	Yes	[92]
		—	—	Yes (101)	12–15 mg·kg^-1^·day^-1,^^e^	Peri-operative	<1–24 months	Cysts	Yes (95)	Mild	[79]
		—	—	Yes (48)	15 mg·kg^-1^·day^-1,^^e^	Pre-operative	4 weeks	N/S	N/S	None	[77]
	AE	Yes	Yes^b^	—	40 μM (*in vitro*)	N/A (*in vitro*)	12 days (*in vitro*)	Cysts	Yes (N/S, *in vitro*)	None	[95]
					200 mg·kg^-1^·day^-1^ (*in vivo*)^e^	8 weeks (*in vivo*)	6 weeks (*in vivo*)		Yes (48, *in vivo*)		
		Yes	Yes^b^	—	20 μM (*in vitro*)	N/A (*in vitro*)	5–10 days (*in vitro*)	Cysts	No (—, *in vitro*)	N/S	[96]
					200 mg·kg^-1^·day^-1^ (*in vivo*)^e^	6 weeks (*in vivo*)	6 weeks (*in vivo*)		Yes (36, *in vivo*)		
		—	Yes^b^	—	200 mg·kg^-1^·day^-1,^^e^	6 weeks	8 weeks	Cysts	Yes (NS)	N/S	[97]
		—	Yes^b^	—	200 mg·kg^-1^·day^-1,^^e^^,^^f^	6 weeks	4–6 weeks	Cysts	Yes (36–84)	N/S	[98]
		—	—	Yes (8)	10–15 mg·kg^-1^·day^-1,^^e^	Variable	6–120 months	Cysts	Yes (38)	Severe	[78]
		—	—	Yes (1)	N/S	At diagnosis	48 months	None	No (—)	Severe	[99]
		—	—	Yes (1)	N/S	At diagnosis	4 months	None	No (—)	N/S	[100]
Albendazole L	CE	Yes	Yes^b^	—	10 μg·mL^-1^ (*in vitro*)	N/A (*in vitro*)	20 days (*in vitro*)	Cysts	No (20, *in vitro*)	N/S	[101]
					75 mg·kg^-1^ (*in vivo*)^e^	6 weeks (*in vivo*)	4 months (*in vivo*)		Yes (85, *in vivo*)		
	AE	—	—	Yes (4)	10 mg·kg^-1^·day^-1.^^e^	Variable	6–120 months	Cysts	Yes (75)	Mild	[78]
Albendazole SD	CE	—	Yes^b^	—	25 mg·kg^-1,^^e^	At infection	30 days	Cysts	Yes (72)	N/S	[27]
		—	Yes^b^	—	25 mg·kg^-1,^^e^	4 months	30 days	Cysts	Yes (91)	N/S	[27]
Albendazole CM	AE	—	Yes^b^	—	150 mg·kg^-1^ 2 days^-1,^^e^	12 weeks	12 weeks	Cysts	Yes (95)	N/S	[103]
Albendazole LNC	CE	—	Yes^b^	—	5 mg·kg^-1^·day^-1.^^e^^,^^j^	5 months	30 days	Cysts	Yes (91^e^, 88^j^)	N/S	[102]
Albendazole sulfoxide	CE	Yes	—	—	50 μg·mL^-1^	N/A	5 min.	PSC	Yes (98)	N/A	[73]
		Yes	—	—	10 nmol·mL^-1^	N/A	30 days	PSC	Yes (50)	N/A	[13]
		Yes	—	—	800 μg·mL^-1^	N/A	10 days	PSC	Yes (100)	N/A	[190]
		—	Yes^b^	—	0.5 mg·kg^-1^·day^-1,^^h^	8 months	15 days	Cysts	Yes (~50)	N/S	[105]
		Yes	—	—	200 μg·mL^-1^	N/A	30 min	PSC	Yes (100)	N/A	[104]
	AE	Yes	—	—	1 μg·mL^-1^	N/A	30 days	PSC	Yes (100)	N/A	[19]
Albendazole sulfoxide SLN	CE	—	Yes^b^	—	0.5 mg·kg^-1^·day^-1,^^h^	8 months	15 days	Cysts	Yes (~37)	N/S	[105]
Albendazole sulfoxide PGLA-PEG	CE	Yes	—	—	200 μg·mL^-1^	N/A	5 min	PSC	Yes (100)	N/A	[104]
Albendazole sulfone	CE	Yes	—	—	50 μg·mL^-1^	N/A	5 min.	PSC	Yes (97)	N/A	[73]
Fenbendazole	AE	Yes	Yes^b^	—	20 μM (*in vitro*)	N/A (*in vitro*)	5–10 days (*in vitro*)	Cysts	No (—, *in vitro*)	N/S	[96]
					200 mg·kg^-1^ day^-1^(*in vivo*)^e^	6 weeks (*in vivo*)	6 weeks (*in vivo*)		Yes (55, *in vivo*)		
Flubendazole	CE	Yes	—	—	10 μg·mL^-1^	N/S	24 days	PSC	Yes (78)	N/A	[112]
		Yes	—	—	10 nmol·mL^-1^	N/S	30 days	PSC	Yes (99)	N/A	[13]
		Yes	—	—	10 μg·mL^-1^	8 months	12 days	Cysts	Yes (N/S)	N/A	[112]
		—	Yes^b^	—	5 mg·kg^-1,^^e^	6 months	25 days	Cysts	Yes (90)	N/S	[13]
		—	Yes^d^	—	10 mg·kg^-1,^^e^	N/A	55 days	Cysts	Yes (82)	N/S	[91]
Flubendazole solution	CE	—	Yes^b^	—	5 mg·kg^-1,^^e^	3 months	25 days	Cysts	Yes (90)	N/S	[113]
		—	Yes^b^	—	5 mg·kg^-1,^^e^	At infection	15 days	Cysts	Yes (77)	N/S	[114]
Flubendazole suspension	CE	—	Yes^b^	—	5 mg·kg^-1,^^e^	3 months	25 days	Cysts	Yes (23)	N/S	[113]
		—	Yes^b^	—	5 mg·kg^-1,^^e^	At infection	15 days	Cysts	Yes (84)	N/S	[114]
Mebendazole OP	CE	—	Yes^b^	—	25 mg·kg^-1,^^e^	8 months	14 days	Cysts	Yes (49–85)	N/S	[68]
Oxfendazole	AE	Yes	—	—	20 μM	N/A	5–10 days	None	No (—)	N/A	[96]
	CE	—	Yes^d^	—	30 mg·kg^-1,^^e^	N/A	11 weeks	Cysts	Yes (50–92)	None	[26]
	AE	—	Yes^d^	—	60 mg·kg^-1,^^e^	N/A	4 weeks	Cysts	Yes (17–40)	None	[108]
Triclabendazole	AE	Yes	—	—	20 μg·mL^-1^	N/A	12 days	Cysts	Yes (100)	N/A	[120]
Triclabendazole sulfoxide	AE	Yes	—	—	20 μg·mL^-1^	N/A	15 days	Cysts	Yes (100)	N/A	[120]

L: Liposomal; N/A: Not applicable; N/S: Not specified; OP: Oil preparation; PSC: Protoscoleces; SD: Solid dispersion; SLN: Solid lipid nanoparticles; CM: chitosan microspheres; LNC: lipid nanocapsules.

^a^ Defined as a significant reduction of the parasite weight, burden or the viability of the any form of the larval stage of the parasite, including protoscoleces or cysts.

^b^ Experimental infection in mice.

^c^ Experimental infection in rabbit.

^d^ Natural infection in sheep.

^e^ Oral or intragastric administration.

^f^ Intraperitoneal administration.

^g^ Intrathoracic administration.

^h^ Intramuscular administration.

^i^ Administration route not specified.

^j^ Subcutaneous administration.

#### Mebendazole

Mebendazole (MBZ) (S1 Fig, compound 1) is a highly effective, broad-spectrum anthelmintic widely used for the treatment of nematodal, cestodal, and even protozoan infections. After its commercialization in 1970, MBZ was the first BMZ agent found to have a lethal effect on *E*. *granulosus* metacestodes in infected patients [46].

Because MBZ is insoluble in water, the drug is poorly available for the treatment of systemic infection such as human CE and AE. The small fraction (1% to 5%) of MBZ absorbed at intestinal level reaches the liver through the hepatic artery and the hepatic portal vein, where is mostly (86%) metabolized to 2-amino-5(6)[a-hydroxybenzyl]benzimidazole. Secondary metabolites include 2-amino-5(6)-benzoylbenzimidazole (13%), methyl-5(6) [a-hydroxybenzyl]benzimidazole carbamate (<1%), and methyl 5(6)-benzoylbenzimidazole carbamate (<1%). All these compounds were thought to be largely inactive [47]. In a group of 12 CE patients orally treated with 10 mg·kg^-1^·day^-1^ MBZ, elimination half-lives ranged from 3–9 h, the time to peak plasma concentration after dosing ranged from 1.5–7 h, and the peak plasma concentrations ranged from 17 to 500 ng·mL^-1^. Additionally, MBZ concentrations in tissue and cyst material at surgery ranged from 60 to 207 ng·g^-1^ wet weight [48]. As a consequence of its poor solubility and bioavailability, MBZ plasma levels are highly variable among patients, as demonstrated in a number of studies [48–51]. Not surprisingly, a direct correlation between the clinical outcome and the MBZ plasma concentration has also been reported in both CE and AE patients [50].

Current recommended dosage of MBZ for the chemotherapeutical treatment of both human CE and AE is 40–50 mg^-1^·kg^-1^·day^-1^ orally in three divided doses during meals [3]. In AE patients MBZ should be administered for at least 2 years after radical surgery, or continuously in inoperable cases, as well as for patients who have undergone incomplete resection or liver transplantation [52]. Long-term MBZ therapy is generally well tolerated, having been used for more than 20 years in some patients [53]. However, mild to serious adverse reactions have been described in 5% to 40% of treated CE or AE patients [54–57]. Reported undesirable side effects included gastrointestinal distress, hair loss, neutropenia, anaphylactic reactions, glomerulonephritis, vertigo, headache, psychic conspicuousness, haematotoxic effects, and abnormal levels of serum transaminases [48,58], with most of these reactions having occurred during the first month of treatment.

Although MBZ during pregnancy hast not been associated with a significantly higher likelihood of developing congenital abnormalities [59], a proper benefit-risk assessment must be conducted when considering treating pregnant or potentially pregnant women, especially in the first trimester. The same considerations should be taken into account with children younger than six years.

During the last three decades, a wealth amount of information has been generated on the efficacy of MBZ therapy for the treatment of human CE and AE. Overall, data from large clinical series have demonstrated that MBZ mainly exerts an inhibitory, rather than curative, effect on the growth of both *E*. *granulosus* and *E*. *multilocularis* metacestodes *in vivo* [57,60,61]. Depending on the *Echinococcus* species involved, the organ affected, the therapeutic regimen, and the methods used for measuring the treatment outcome, MBZ chemotherapy was successful in 14% to 49% of cases, whereas improvement was observed in 29% to 70% of the treated patients. In 16% to 43% of cases the MBZ treatment failed [57,61–64]. Interestingly, MBZ therapy success has been shown to be greatly influenced by cyst size and organ affected. Thus, MBZ treatment was more effective in cysts whose mean size was ≤ 5 cm, whereas it was largely unsuccessful in cysts with a mean size of ≥ 7 cm [64]. Similarly, in a case series of 23 patients with CE or AE, cysts regression was more commonly observed in pulmonary (83%) than in hepatic (18%) locations [65].

In recent years little research has been directed towards the improvement of the MBZ chemotherapeutical activity. This is in large part due to the progressive substitution of MBZ by albendazole in the treatment of human CE and AE (see below). Efforts have mainly been done to develop new formulations to enhance the solubility and oral bioavailability of the drug. In this regard, micro-particles of hydroxypropylcellulose, a cellulose derivative polymer used for the preparation of artificial tears in the ophthalmic industry, has recently been used as a carrier of MBZ at low dose (5 mg·kg^-1^) [66]. This formulation proved significantly more active than pure MBZ against all the developmental stages of the nematode *Trichinella spiralis* in a murine model. Similarly, cyclodextrins plus ABZ were more active than pure ABZ against this nematode [67]. This system may represent a more secure drug delivery system in the clinical treatment of systemic helminthic infections. Based on early experiments showing that oral administration of MBZ with olive oil slightly increase its bioavailability [47], nine different MBZ-oil preparations were used to assess the efficacy of these formulations in mice with experimentally induced secondary hydatidosis. Among them, oleic acid, glycerol trioleate, and soybean oil tested at 25 mg·kg^-1^ achieved the best results. Compared to controls, these three MBZ-oil preparations evidenced a 1.6–2.8-fold increase in plasma concentration, a 1.7–2.4-fold increase in bioavailability, and a 1.8–3.3-fold reduction in cyst weight. Independently of the oil formulation used, MBZ levels in plasma were maintained for about 15 h post-administration [68] (Table 1).

#### Albendazole

Albendazole (ABZ) (S1 Fig, compound 2) is a BMZ derivative with a broad-spectrum activity, including helminthic and protozoan infections. First introduced for human use in 1982, ABZ has now replaced MBZ as the drug of choice for the treatment of CE and AE, due mainly to its improved bioavailability, superior efficacy, easier administration, and lower undesired effects [4,57,62,69]. In addition, ABZ is 40% cheaper than MBZ [58]. Still, ABZ availability and/or cost continue to be an issue in many socioeconomically disadvantaged and even in high-income countries [70].

Because ABZ is poorly absorbed in the gastrointestinal tract after oral administration, higher doses of the parent drug are required to elicit effective therapeutic responses at the tissue level. After intestinal absorption, ABZ passes on to the bloodstream and is carried to the liver, where it is extensively metabolized. The process is so rapid that plasma levels of the parent drug are usually undetectable [71]. ABZ is then oxidized to ABZ sulfoxide (S1 Fig, compound 3) (ABZSO), also known as ricobendazole, the main metabolite *in vivo* [72]. ABZSO can be further oxidized to ABZ sulfone (S1 Fig, compound 4) (ABZSN), a metabolite that has demonstrated *in vitro* activity against *E*. *granulosus* protoscoleces and *E*. *multilocularis* vesicles [73,74]. In an early study assessing the pharmacokinetics of ABZ in 11 pre-surgical CE patients, ABZSO plasma levels remained stable (0.6–1 μg·mL^-1^) after 2 to 4 days of treatment, whereas intra-cyst concentration was 0.921 μg·mL^-1^ [75]. Similar results were obtained in two CE patients treated with ABZ for 4–5 days before surgical removal, with pre- and intra-operative plasma levels of ABZSO ranging from 0.18–1.26 μg·mL^-1^ to 0.10–1.24 μg·mL^-1^, respectively. Intra-cyst concentrations of ABZSO were estimated at 0.16 μg·mL^-1^ and 0.59 μg·mL^-1^, respectively [76]. In a recent survey examining 48 CE patients treated with 15 mg·kg^-1^·day^-1^ ABZ for 10 days before surgery ABZSO concentrations were measured both in plasma (0.431 μg·mL^-1^) and cysts (0.279 μg·mL^-1^) at the moment of surgical intervention. In this study no clear association between plasma and cyst ABZSO concentration could be demonstrated, maybe due to the different cyst types detected in the patients’ cohort [77]. *In vitro* parasitocidal activity of ABZSO against *E*. *multilocularis* vesicles has been found at 10 μg·ml^-1^ in the culture medium, reaching 2.5 to 5.5 μg·ml^-1^ inside the vesicles [74], a concentration three to ten times higher than that reported inside CE cyst of ABZ treated patients.

ABZ oral administration is currently recommended at a dosage of 10–15 mg^-1^·kg^-1^·day^-1^ in two divided doses for the treatment of both CE and AE [3]. Because ABZ is nowadays considered a relatively safe drug, continuous therapy is preferred over discontinuous treatment protocols. Recent descriptive studies based on case series have evidenced that the frequency of undesirable side effects attributable to ABZ ranged from 3–5% [58,78,79], with some surveys reporting none [80]. The adverse reactions more frequently described were jaundice, severe headache, cough, hemoptysis, altered levels of serum transaminases, vertigo, loss of hair, and itching [58,78,79]. Despite the fact that ABZ and ABZSO have been demonstrated teratogenic in rats and rabbits at 6–30 mg^-1^·kg^-1^·day^-1^, examination of 49 case histories of women prescribed with ABZ for the treatment of various helminthic infections during the first trimester of pregnancy failed to demonstrate congenital abnormalities associated to drug exposure [81]. Additionally, and due to the scarcity of CE and AE cases in patients of pediatric age, ABZ has not been fully evaluated in children younger than 6 years of age. However, it is important to take into account that no proper pharmacokinetics studies have been conducted to conclusively validate the optimal time course and ABZ dosage for the treatment of human CE and AE. This is mostly because the clinical management of CE and AE performed by different centres globally is extremely heterogeneous. In addition, the slow progress of these diseases together with the relatively low number of confirmed cases even in endemic areas makes very difficult the design and implementation of clinical trials [82].

As previously mentioned poor water solubility and weak intestinal absorption of ABZ compromises its therapeutic effectiveness due to low bioavailability both in plasma and intra-cyst. In an attempt to overcome this situation, a number of strategies have been tested to enhance the bioavailability of the drug. For instance, it has been demonstrated that plasma concentration of ABZSO was 4.5-fold higher when ABZ was given with a fatty meal than when administered in the fasting state [83]. Chemical modifications of the parent compound have also been assessed. In this regard, an ester of ABZ has been synthesized with significant improved solubility. This ABZ derivative exhibited a similar scolicidal activity than that observed for the parent compound in *in vitro* experiments [84]. Toxicological tests conducted in mice orally or intraperitoneally administered with the drug showed little effect, although more research is needed to ascertain the efficacy of this chemically modified compound in experimental animal models.

A significant amount of effort has also been devoted to develop novel formulations and technologies for delivering ABZ into tissues in order to enhance its therapeutic effects. In this regard, of particular interest is the appraisal of sustained release formulations, including liposomes, biodegradable microspheres, and polymer conjugates, targeting specific organs in which the drug is released over a period of time in a controlled manner. I*n vitro* experiments with ABZ-encapsulated conventional liposomes and polyethylene glycol (PEG)-coated liposomes have demonstrated release of the drug for prolonged periods of time when compared with the parent compound [85]. Similarly, ABZ matrix tablets containing synthetic polymers have been used to mimic *in vitro* the release of ABZ in fresh caecal content obtained from euthanized rats [86].

More physiologically relevant data comes from an *in vivo* rat model evaluating the effectiveness of ABZ associated to nanoparticles of the linear polysaccharide chitosan as a liver-targeting delivery system [87]. This formulation also included Poloxamer 188 as auxiliary solvent to increase ABZ solubility and enhance its absorption at the intestinal level. As demonstrated by *in vivo* near-infrared fluorescence real-time imaging, chitosan nanoparticles succeeded in accumulating both ABZ and ABZSO in the liver of orally administered rats at concentrations much higher than that obtained using MBZ as internal control [87]. Additionally, this formulation combined also efficient drug loading, high physical stability, and low toxicity, features that made it a promising delivery system for ABZ in the treatment of human CE and AE. Finally, clinical data on the efficiency of formulations based on ABZ carrier vehicles is very limited. Thus, in a recent case series three out of four (75%) patients who suffered from late-stage AE and were administered with 10 mg·kg^-1^·day^-1^ liposomal ABZ achieved clinical improvement, as determined by imaging methods confirming cyst regression and clinical manifestation evaluation [78]. Although indicative of a parasitostatic rather than a parasitocidal effect, this success rate was significantly higher than that (38%) obtained with ABZ administered as parent compound, suggesting that liposomal formulation considerably improved the bioavailability of the drug. In addition, liposomal ABZ therapy was associated with milder undesirable side effects compared with ABZ.

In recent years the chemotherapeutical efficacy of ABZ and its derivatives against the larval stages (including protoscoleces) of *E*. *granulosus* and *E*. *multilocularis* has been assessed in a number of *in vitro* and *in vivo* studies (Table 1). In this regard, it is worth to mention that direct comparison among results obtained from different research groups is difficult due to the lack of standardized protocols and procedures.

ABZ at 10 nmol·mL^-1^ was demonstrated to reduce 70% the vitality of *E*. *granulosus* protoscoleces in culture after 30 days of incubation [13]. Experimental infection of mice with protoscoleces of *E*. *granulosus* constitutes a widely used *in vivo* model for the assessment of ABZ efficacy against secondary hydatidosis [13,28]. ABZ treatment for 20–30 days at different dose regimes had both chemo-prophylactic and therapeutic properties in infected mice, achieving a higher reduction in cyst weight compared to control (untreated) mice [27,88,89]. ABZ efficacy was further enhanced (91–98%) when prepared as solid dispersion in formulation with Poloxamer 188 [27]. In addition, 150 mg·kg^-1^ ABZ administered for 10 consecutive days from the time of infection was demonstrated to completely inhibit cyst growing [88]. ABZ has also been tested in the treatment of secondary pleural hydatidosis in rabbits [90]. In this animal model intra-thoracic administration of 2% ABZ as a single dose was simultaneously conducted with the injection of hydatid cyst fluid containing fertile protoscoleces into the pleural cavity of experimentally infected rabbits. The chemo-prophylactic efficacy of the treatment was demonstrated by the failure to develop hydatid cysts in treated animals compared to controls. Oral treatment with ABZ over 55 days was also effective against the larval stage of *E*. *granulosus* in naturally infected sheep [91]. In this study, the vitality and *in vivo* development to cysts in mice of protoscoleces obtained from hydatid cysts recovered from treated animals was greatly reduced compared to protoscoleces obtained from unmedicated animals.

ABZ has been the drug of choice for the treatment of human CE cases in later years. Therefore, a number of clinical series studies have been recently conducted to assess the therapeutic effect of ABZ either in long-term treated patients [80] or as a complement to surgical procedures to prevent recurrence [77,79,92]. Depending on the study duration of treatment was highly variable, ranging from less than a week to 24 months. In all cases ABZ dosages used were within the recommended range. Importantly, the final efficacy assessment of ABZ may vary in function of the criteria chosen to define success between treatment outcomes. For instance, when this parameter was defined as the complete disappearance of all cysts or a reduction of cyst size ≥ 25% after treatment, the efficacy of ABZ therapy was estimated in the range of 26–34% in a case series of Peruvian patients [80]. However, higher success rates may be reported if less stringent definitions are adopted [93,94]. In addition, intra- and peri-operative treatment with ABZ has been demonstrated effective to prevent recurrence following surgical or percutaneous (PAIR) procedures [79,92]. In this regard, ABZ treatment initiated 1 week before and continued for 1 month after PAIR procedure has been shown to be sufficient to reduce or even prevent recurrence of the infection [92]. It should be also noted that length of ABZ therapy was not necessarily associated with satisfactory treatment outcome, as evidenced in some long-term and follow-up studies [80], as the success/failure balance is also influenced by a number of factors, including the size, location, and type of the cyst, or the age and overall health status of the patient. Overall, prescribed ABZ regimes were well tolerated, even in long-term therapies. When present, undesirable side effects were mild and rarely treatment-limiting [79].

As in the case of CE, ABZ is also the preferred drug for the treatment of human AE nowadays. *In vitro* treatment of *E*. *multilocularis* metacestodes with 40 μM ABZ for 12 days led to a rapid increase of alkalin phosphatase EmAP (a marker of pharmacological damage in treated metacestodes) activity within four days of culture [95]. However, a lower concentration of the drug had no effect in parasite vesicles treated for a shorter period of time, based on the measurement of PGI activity as viability marker [96]. Studies relying on experimentally infected mice developing secondary AE have also been used as *in vivo* models to assess the efficacy of ABZ against the parasite. Oral or intra-gastric administration of the drug at 200 mg·kg^-1^·day^-1^ for 6–8 weeks have demonstrated a reduction in parasite weight in the range of 36% to 48% in treated mice compared to the untreated control group [95–98]. Interestingly, intra-peritoneal administration of 200 mg·kg^-1^·day^-1^ ABZ for four weeks was far more effective, achieving a reduction of 84% in the parasite burden [98], although the translation of this regime to human treatment in not very feasible. In a case series of eight AE patients with multi-organ involvement including liver, lung, and brain, 10–15 mg·kg^-1^·day^-1^ ABZ therapy showed a 38% cure rate [78] well in line with previously published data [4,58]. However, severe clinical symptoms including jaundice, cough and hemoptysis were reported in some of the treated patients. Although ABZ is capable of exerting a true parasitocidal effect, treatment discontinuation often leads to the recurrence of parasite growth [99,100], maybe related with the more frequent parasitostatic effect *in vivo* or to other reasons, e.g. the tubulin insoform expressed by the parasite’s stem cells show limited affinity to BMZ [31].

In an attempt to improve ABZ solubility and subsequent bioavailability liposomal formulations of the drug have been tested for chemotherapy against both CE and AE. Although liposomal ABZ treatment of *E*. *granulosus* protoscoleces cultured *in vitro* only showed a very limited (~20%) effect in viability reduction, intra-gastric administration of the same formulation in mice with secondary hydatidosis was sufficient to prevent cyst development in 17 out of 20 (85%) treated mice [101]. Unfortunately, pure ABZ was not used as a control in this study, so a direct comparison of the therapeutic effectivities of both compounds was not possible. Liposomal ABZ formulations have also been successfully assessed against human AE. Thus, in a clinical series of four AE patients with multi-organ involvement administration of this formulation significantly improved the therapeutic efficacy of conventional ABZ treatment from 38% to 75%. Additionally, liposomal ABZ-treated patients experienced lesser and milder clinical symptoms compared to conventional ABZ-treated patients. [78].

A drawback of liposomes is their limited loading capacity of hydrophobic drugs, including ABZ. As an alternative, lipid nanocapsules are used to encapsulate lipophilic drugs without using organic solvents. A formulation in lipid nanocapsules to enhance ABZ bioavailability was described and tested against secondary CE in mice, showing 91% cyst weight reduction and enhancing the results obtained when animals were treated with ABZ (47% reduction) [102]. ABZ has been also formulated in chitosan microspheres [103]. These have shown effectivity against AE secondary infection in mice after oral administration, resulting in 94.5% of cyst weight reduction in treated mice, in comparison with 78.5% and 91.2% reduction achieved after treatment with ABZ and ABZ liposomes, respectively [103]. When ABZ sulfoxide concentration profiles were compared, these were enhanced in mice treated with the microspheres compared with those treated with liposomes. Moreover, treatment with ABZ in chitosan microspheres induced a shift from Th2 to Th1 (potentially protective) response in mice. A further advantage of those microspheres compared with liposomes is their stability and simpler manufacturing [103].

As previously commented, ABZSO (S1 Fig, compound 3) is the main active metabolite of ABZ *in vivo* [72]. This ABZ derivative has also evidenced a potent scolicidal activity *in vitro*. For instance, 50 μg·mL^-1^ ABZSO was capable to kill 98% of *E*. *granulosus* protoscoleces in only 5 min. Very similar results were also obtained under the same treatment conditions with ABZSN (S1 Fig, compound 4), an ABZ derivative metabolite initially thought to be inactive [73]. In another study protoscolex viability decreased to ~45% after 30 days of incubation when a much lower dose of 10 nmol·mL^-1^ (equivalent to 2.6 ng·mL^-1^) ABZSO was used, a dose closer to the intra-cyst concentrations found in treated patients than those in the range of μg·mL^-1^ [13]. ABZSO was compared to ABZSO loaded in PGLA-PEG nanopolymeric, long circulating particles against *E*. *granulosus* protoscoleces *in vitro*, achieving 100% scolicidal effect with 200 μg·mL^-1^ in 5 min, six times faster than with ABZSO alone [104]. Similarly, ABZSO at 1 μg·mL^-1^ concentration rendered inactive 100% of *E*. *multilocularis* protoscoleces after a 30 days exposure [58]. In addition, a recent *in vivo* study in mice with experimentally induced secondary CE has demonstrated that intramuscular administration of ABZSO at low dose (0.5 mg·kg^-1^·day^-1^) led to a~50% reduction in cyst size and weight in comparison to those collected from untreated control mice. Use of ABZSO at the same concentration but formulated as solid lipid nanoparticles did not further improve these results [105].

#### Fenbendazole

Since its introduction in 1974 fenbendazole (FBZ) (S1 Fig, compound 5) has been shown as a broad spectrum ABZ anthelmintic effectively used against gastrointestinal parasites including nematodes, cestodes belonging to the genus *Taenia*, and some protozoan species such as *Giardia*. FBZ has been extensively used in veterinary medicine, but it is not currently licensed for human use.

The mechanism of action of FBZ is as other members of the BMZ family [35], and also has a very poor solubility in water, so oral administration of the drug in production animals leads to a limited absorption into the bloodstream. Absorbed FBZ is metabolised in the liver to its active sulfoxide derivative, which is identical with oxfendazole (see below), and subsequently to its sulfone derivative [106].

As in the case of ABZ, treatment of *E*. *multilocularis* metacestodes with FBZ *in vitro* was ineffective in inducing damage in the parasite´s vesicles, as no measurable levels of the PGI marker could be detected [96]. However, experimentally infected mice treated with FBZ exhibited a 55% reduction in parasite burden compared with untreated control mice. In the same study, ABZ-treated mice under the same treatment regimen only achieved a 36% reduction in parasite weight. Whether the activity of FBZ *in vivo*, compared with its lack of activity *in vitro*, could be attributed to a concomitant action of the immune response of the host, should be investigated. Given these results, the authors proposed the use of FBZ as an alternative chemotherapeutical agent for the treatment of human AE.

#### Oxfendazole

Oxfendazole (OXF) (S1 Fig, compound 6) is the sulfoxide metabolite derivative of FBZ. This compound is much less frequent used in veterinary medicine than its parent molecule. Its use has not been licensed for human use. As also demonstrated for both ABZ and FBZ, OXF treatment had no apparent effect on *E*. *multilocularis* metacestodes cultured *in vitro* [96]. The chemotherapeutical efficacy of OXF has been evaluated in a number of production animal species naturally infected with *E*. *granulosus*. Suprisingly, many of those *in vivo* studies refer to the effect of OXF in protoscolex viability, but not to its effects in cyst reduction or viability. In an early study, absent or dead protoscoleces were found in 97% of cysts isolated from OXF-treated animals compared to 28% of cysts from untreated control animals. Although the authors claimed that OXF was at least as effective as ABZ for the treatment of CE, no direct comparison between the two BMZ compounds was carried out [107]. Somewhat similar results were obtained in a field trial survey where 30 sheep with CE were orally treated with OXF. After necropsy, OXF treatment induced a viability reduction of 50% and 92% in protoscoleces collected from lung and liver cysts, respectively, in treated animals compared to untreated control animals. Not surprisingly, OXF-treated sheep were associated with a mean weight gain > 3 kg compared to control animals. No side effects were reported with this treatment regimen [26]. In a following study, OXF administered to sheep naturally infected with *E*. *granulosus* was effective in reducing the mean diameter or lung and liver cysts by 17% and 40%, respectively, compared to placebo [108]. These results support that OXF should be further tested *in vivo* ideally compared with ABZ, regarding both activity and side effects, to guarantee its use in patients.

#### Flubendazole

Initially developed in the 1970s flubendazole (FLBZ) (S1 Fig, compound 7), the ρ-fluoro analogue of MBZ, is a synthetic broad-spectrum anthelminthic widely used in veterinary medicine, particularly in poultry and swine. FLBZ is currently registered in Europe as an anthelmintic in humans for intestinal nematodes including pinworms (*Enterobius vermicularis*), roundworms (*Ascaris lumbricoides*), hookworms *(Ancylostoma duodenale*) and whipworms (*Trichuris trichiura*). Complying with the inherent physicochemical features of BMZ, FLBZ has poor solubility and very low bioavailability, as demonstrated in studies of the *in vivo* biotransformation of FLBZ administered to production animal species. Thus, intra-ruminal administration of FLBZ showed low plasma concentrations of FLBZ parent drug being measured between 6 and 48 h, and only trace concentrations of hydrolysed FLBZ being detected [109]. Subsequent research has demonstrated that the metabolic biotransformation of FLBZ into reduced FLBZ (R-FLBZ) was taking part in the microsomal and cytosolic subcellular fractions obtained from sheep liver and duodenal mucosa, with most (~75%) of R-FLBZ being synthesized at the hepatic level and the remaining fraction being metabolized at the intestinal level [110].

The therapeutic properties of FLBZ against *Echinococcus* infection have been only attempted in a limited number of studies. Revealingly, treatment of *E*. *granulosus* protoscoleces with FLBZ resulted in marked transcriptional modifications of structural and metabolic genes essential for the energy production metabolism of the parasite that were not observed when ABZ or nitazoxanide were used at the same concentration [111]. More specifically, FLBZ treatment induced a 90% reduction in tubulin transcripts (as determined by RT-PCR and pPCR) after 72 h post-treatment, compared to untreated controls. This effect was paralleled by a significant (~80%) decrease in the gene expression and activity of the malate dehydrogenase. In addition, after 24 h exposure to FLBZ a transient increase of intracellular Ca^2+^ followed by a slow recovery to basal levels was observed. Because this intracellular Ca^2+^ raise took part before the depletion of cellular glycogen storages, the authors suggested that Ca^2+^ levels may play a key role in the downstream signalling pathways controlling the energy metabolism of the parasite [111].

FLBZ at concentrations in the nanomolar range has evidenced a potent scolicidal activity (78–99%) *in vitro*, but at exposure times ranging from 24 to 30 days [13,112]. Interestingly, *E*. *granulosus* protoscoleces incubated with 10 nmol·mL^-1^ FLBZ exhibited the quickest reduction in protoscoleces viability compared to those obtained after treatment with the same concentrations of ABZ, ABZSO, and R-FLBZ [13]. Additionally, experimentally infected mice have been used as *in vivo* model for the assessment of the therapeutic properties of FLBZ against *E*. *granulosus*. Initial experiments using FLBZ orally administered managed to reduce 90% the mean cyst weight of FLBZ-treated mice compared to untreated infected controls [13]. In treated infected mice, FLBZ and R-FLBZ achieved peak plasma concentrations of 1.56 μg·mL^-1^ and 1.02 μg·mL^-1^, respectively, at 30–40 min after the administration of the parent compound, being detected up to 6 h post-treatment [13]. Further studies using a double FLBZ dosage and a longer exposure time did not further improve this success rate [91]. In an attempt to enhance FLBZ bioavailability and *in vivo* efficacy, this drug has been experimentally assessed in solution containing hydroxipropyl-β-cyclodextrin or in suspension containing carboxymethyl cellulose. Oral administration of FLBZ formulated as a solution to healthy mice resulted in FLBZ plasma concentrations higher than those measured for its reduced or hydrolysed metabolites, peaking at 42 min post-treatment, and much higher than that reached after the oral administration of FLBZ formulated as a suspension [113]. In addition, after administration of FLBZ-solution to mice with secondary CE, the mean cyst weight was reduced by 78% three months post-infection, compared to the values obtained for cysts recovered from untreated mice. Treatment with FLBZ-suspension did not achieve a significant reduction of the parasite burden [113]. Interestingly, both FLBZ-solution and FLBZ-suspension formulations exhibited a strong chemoprophylactic activity when orally administered to mice experimentally infected by intraperitoneal inoculation with *E*. *granulosus* protoscoleces. This treatment regimen suffices to reduce by 77–84% the mean cyst weight of treated mice in comparison to unmedicated control mice [114]. The study of the efficacy of FLBZ and their derivatives for the treatment of AE has not been attempted to date. Among the recently developed new strategies aiming to improve FLBZ systemic availability after oral administration, the use of amorphous solid dispersion (ASD) formulations is one of the most promising, as evidenced by the encouraging results obtained in murine efficacy models of filariasis [115]. ASD-based preparations still have not been evaluated for the treatment of human CE and AE.

#### Triclabendazole

Triclabendazole (TCBZ) (S1 Fig, compound 8) is a narrow-spectrum BMZ compound mainly used to treat human cases of fasciolosis, although it is not currently licensed for the management of human CE and AE. From a molecular point of view, the chemical structure of TCBZ differs from other members of the BMZ family in which it contains a chlorinated benzene ring but no carbamate group, features that may well account for the highly specificity of TCBZ for liver flukes, with little or still unclear activity against nematodes, cestodes, and other trematodes. TCBZ has been demonstrated as a potent inhibitor of protein synthesis [116], and the ATP-binding cassette transporter ABCG2/BCRP, a membrane-associated protein involved in the extrusion of a wide range of xenotoxins [117], in addition to the customary microtubule-inhibiting mode of action of all BMZ members. After oral administration, TCBZ is rapidly absorbed at the intestinal level. In sheep, biotransformation of the parent compound in liver microsomes resulted in the generation of the active metabolite TCBZ-sulfoxide (TCBZSO) and thereafter to TCBZ-sulfone (TCBZSN) and hydroxy derivatives [118,119].

So far, the chemotherapeutical properties of TCBZ against *Echinococcus* has been only tested in *in vitro*-cultured *E*. *multilocularis* metacestodes, where incubations with 20 μg·mL^-1^ TCBZ or TCBZSO for 12 days or 15 days, respectively, caused a complete disruption of the parasite´s vesicles [120]. Further *in vivo* studies should be done for its recommendation for the potential treatment of AE and CE patients. In this sense, recommended TCBZ dosages for the treatment of human fascioliasis is similar to those recommended for other BMZ compounds used against AE and CE [121]. Reported adverse reactions associated to TCBZ therapy are also similar to those reported for other BMZ. No information is currently available regarding the potential teratogenicity of TCBZ in pregnant women, or its suitability for the treatment of patients of paediatric age.

#### Sodium salts of ricobendazole and triclabendazole sulfoxide

Anthelmintic compounds containing a sulfanyl-benzimidazole (SBZ) scaffold such as ABZ and TCBZ are *in vivo* oxidized into sulfinyl-benzimidazole (SOBZ) derivatives (ricobendazole, RBZ, and triclabendazole sulfoxide, TRBZSO, respectively) which are the active metabolites of drugs. The sulfur atom of the sulfoxide group is a stereogenic center and, consequently, RBZ, and TRBZSO consist of an equimolar mixture of two enantiomers. *In vivo* studies have highlighted a stereoselectivity in SOBZ drug-organism interaction depending on the species, age and gender of the host as well as the type of parasite [122–124]. As an example, following oral administration of RBZ, it has been demonstrated that the (−)-RBZ enantiomer is predominant in rats and mice, whereas the (+)-RBZ enantiomer is prevalent in sheep, goats, dogs, cattle and humans [125]. The different plasma concentration of the two enantiomers suggests a potential therapeutic application of enantiopure forms of sulfoxides. As above mentioned, ABZ and TCBZ are totally insoluble in water whereas RBZ and TRBZSO are poorly soluble in aqueous conditions and in most of the injectable co-solvents or surfactants.

Recently, a simple synthetic strategy based on the presence of the acidic NH group in the benzimidazole nucleus has been presented with aim to obtain new water-solubility salts of RBZ and TRBZSO [126,127]. Accordingly, the nitrogen atom of the imidazole nucleus has been successfully deprotonated and salified through the addition of freshly ground sodium hydroxide at room temperature. The transformation of racemic or enantiopure sulfoxides, which can be obtained on a semipreprative scale by enantioselective HPLC on polysaccharide-based chiral stationary phases, into their sodium salts (RBZ-Na and TRBZSO-Na) offers the opportunity to optimize the physicochemical parameters of the ionizable drug molecules without changing their pharmacologically active moiety. Since the single synthetic step affords quantitative yields and uses ethanol as solvent, the proposed approach seems to be ideally suited for a sustainable large-scale pharmaceutical development of the alkaline salts.

Dissolution experiments via spectrophotometric measurements indicate that RBZ-Na salt is highly soluble either in water and 0.01 M phosphate pH 7.4 buffer solution. In fact, the solubility of RBZ-Na is 22.83 mg·mL^−1^ in water (*vs* 0.06 mg·mL^−1^ of the unsalified form) and 14.49 mg·mL^−1^ at physiological pH [126]. Because of these encouraging results, the preparation of injectable anthelmintic formulations based on both racemic and enantiopure forms of RBZ-Na seem to be potentially possible. More recently, efficacy of RBZ-Na and its enantiomers [(*R*)-RBZ-Na; (*S*)-RBZ-Na)] has been tested in a BALB/C model of experimental secondary CE, resulting in extensive damage to the parasite in preclinical studies (Casulli, personal communication).The novel sodium salt of TRBZSO has been also subjected to solubility tests using as dissolving medium a 0.01 M phosphate buffer solution at pH 7.4. Unlike TRBZSO which is practically insoluble at physiological pH (i.e. more than 100 mL of solvent are needed to dissolve 0.5 mg of sulfoxide), TRBZSO-Na exhibits the notable solubility of 19.3 mg·mL^−1^ [127].

As a general principle, it should be stressed that attempting to improve systemic bioavailability of BMZ drugs can results in an increase of side adverse events that could be overcome by decreasing currently recommended human dosage.

#### Benzimidazoles in combined therapies with other biocidal agents

In an attempt to improve the chemotherapeutic management of human CE and AE, a number of BMZ agents have been assessed in combined treatment regimens with other drugs including synthetic and natural compounds [39,42,43]. The rationale behind this strategy is based on the ground that interaction between two drugs may well have a synergistic effect resulting in a significant enhancement of their individual therapeutic performances. In this regard, relevant *in vitro* and *in vivo* data concerning the combined chemotherapeutic efficacy of BMZ with other anti-infective agents for the treatment of CE and AE reported in the literature from 2008 to date are shown in Table 2. Some of these compounds have also been used alone, and as such are also revised in their respective sections.

**Table 2:** Characteristics of benzimidazole compounds, including their metabolites and solubility-improved formulations, in combination with other drugs for the treatment of human cystic and alveolar echinococcosis based on the published literature from 2008 to date.

Compound	Disease	Assay setting			Dosage	Treatment		Efficacy assessment			Reference
		*In vitro*	*In vivo*	In patients (*n*)		Initiation	Duration	Activity against	Success^a^ (%)	Toxicity	
Albendazole + Amphotericin B	AE	Yes	—	—	1 μg·mL^-1^ (ABZSO) + 2.5 μg·mL^-1^ (AMB)	N/A	16 days	Cysts	Yes (100)	N/A	[19]
Albendazole + Artesunate	AE	—	Yes^b^	—	50 mg·kg^-1^·day^-1^ (ABZ)^d^ + 200 mg·kg^-1^·day^-1^ (Artesunate)^d^	8 weeks	6 weeks	Cysts	Yes (76)	Yes	[95]
Albendazole + DHA	AE	—	Yes^b^	—	50 mg·kg^-1^·day^-1^ (ABZ)^d^ + 200 mg·kg^-1^·day^-1^ (DHA)^d^	8 weeks	6 weeks	Cysts	Yes (76)	Yes	[95]
Albendazole L + Huaier	CE	Yes	Yes^b^	—	*In vitro*: 10 μg·mL^-1^ (ABZ L) +2 mg·mL^-1^ (Huaier)	N/A (*in vitro*)	12 days (*in vitro*)	PSC, Cysts	Yes (~50, *in vitro*)	N/S	[101]
					*In vivo*: 75 mg·kg^-1^ (ABZ L)^d^ + 15 g·kg^-1^ (Huaier)^d^	6 weeks (*in vivo*)	4 months (*in vivo*)		Yes (95, *in vivo*)		
Albendazole + Lufenuron	CE	—	Yes^b^	—	50 mg·kg^-1^ (ABZ)^d^ + 100 mg·kg^-1^ (LFR)^d^	4 months	45 days	Cysts	Yes (28–38)	N/S	[28]
Albendazole L + Mefloquine	AE	—	Yes^b^	—	200 mg·kg^-1^·day^-1^ (ABZ)^d^ +25 mg·kg^-1^ (MFQ)^d^	6 weeks	8 weeks	Cysts	Yes (N/S)	N/S	[97]
Albendazole + Metformin	CE	—	Yes^b^	—	5 mg·kg^-1^·day^-1^ (ABZ)^d^ + 50 mg·kg^-1^·day^-1^ (MET)^d^	4 months	8 weeks	Cysts	Yes (96)	N/S	[129]
Albendazole + Praziquantel	CE	—	Yes^c^	—	30 mg·kg^-1^ (ABZ)^d^ + 40 mg·kg^-1^ (PZQ)^d^	N/A	6 weeks	Cysts	Yes (70)	None	[108]
		—	—	Yes (57)	400 mg·day^-1^ (ABZ)^d^ + 20–75 mg·kg^-1^ (PZQ)^d^	Unknown	1–109 months	Cysts	Yes (~45)	Mild	[130]
Albendazole + Thymol	AE	Yes	Yes^b^	—	*In vitro*: 10 μg·mL^-1^ (ABZ) + 10 μg·mL^-1^ (THY)	N/A (*in vitro*)	18 days (*in vitro*)	PSC, Cysts	Yes (33, *in vitro*)	N/S	[131]
					*In vivo*: 5 mg·kg^-1^ (ABZ)^d^ + 40 mg·kg^-1^ (THY)^d^	7 weeks (*in vivo*)	20 days (*in vivo*)		Yes (83.5, *in vivo*)		[132]
		Yes	—	—	10 μg·mL^-1^ (ABZ) +10 μg·mL^-1^ (THY)	N/A	10 days	Cysts	Yes (N/S)	N/A	[131]
Albendazole sulfoxide + Metformin	CE	Yes	—	—	2.5 μM (ABZSO) + 1mM (MET)	N/A	7 days (PSC), 27 days (Cysts)	PSC, Cysts	Yes (100)	N/A	[129]
Albendazole sulfoxide + sulfone	CE	Yes	—	—	50 μg·mL^-1^ (ABZSO) + 50 μg·mL^-1^ (ABZSN)	N/A	5 min.	PSC	Yes (99)	N/A	[73]
Flubendazole + Ivermectin	CE	Yes	—	—	10 μg·mL^-1^ (FBZ) + 1 μg·mL^-1^ (IVM)	N/A	24 days	Cysts	Yes (96)	N/A	[112]
		Yes	—	—	1 μg·mL^-1^ (FBZ) + 1 μg·mL^-1^ (IVM)	N/A	12 days	Cysts	Yes (N/S)	N/A	[112]
Flubendazole + Nitazoxanide	CE	Yes	Yes^b^	—	10 μg·mL^-1^ (FBZ) + 10 μg·mL^-1^ (NTZ)	N/A (*in vitro*)	12 days (*in vitro*)	PSC, Cysts	Yes (100, *in vitro*)	N/S	[133]
					10 mg·kg^-1^ (ABZ)^d^ + 100 mg·kg^-1^ (NTZ)^d^	8 months (*in vivo*)	25 days (*in vivo*)		Yes (66, *in vivo*)		
Oxfendazole + Nitazoxanide	CE	—	Yes^c^	—	30 mg·kg^-1^ (OXF)^d^ + 15 mg·kg^-1^ (NTZ)^d^	N/A	11 weeks	Cysts	Yes (61–100)	None	[26]
Oxfendazole + Praziquantel	CE	—	Yes^c^	—	30 mg·kg^-1^ (OXF)^d^ + 40 mg·kg^-1^ (PZQ)^d^	N/A	6 weeks	Cysts	Yes (72)	None	[108]

ABZ: Albendazole; ABZSN: Albendazole sulfone; ABZSO: Albendazole sulfoxide; AMB: Amphotericin B; DHA: Dihydroartemisin; IVM: Ivermectin; LFR: Lufenuron; NTZ: MFQ: Mefloquine; Nitazoxanide; OXF: Oxfendazole; PZQ: Praziquantel; PSC: Protoscoleces; THY: Thymol

^a^ Defined as a significant reduction of the parasite burden or the viability of the any form of the larval stage of the parasite, including protoscoleces or cysts.

^b^ Experimental infection in mice.

^c^ Natural infection in sheep.

^d^ Oral or intra-gastric administration.

Because ABZ is by far the drug most frequently used in clinical treatment regimens nowadays, it is not surprising that this compound is usually the most frequently used drug in combined therapies. For instance *in vivo* treatment of *E*. *multilocularis*-infected mice with ABZ in combination with its derivative dihydroartemisinin (see below) has been demonstrated effective in reducing by 76% the parasite weights compared to untreated control mice. Under the same experimental conditions, ABZ monotherapy achieved a 48% reduction in cyst weight. Importantly, none of the treated mice exhibited aberrant behaviour or abnormal histopathological findings, suggesting that treatments were well tolerated [95].

Intra-gastric administration of ABZ formulated as a liposomal preparation in combination with Huaier (*Trametes robiniophila*) aqueous extract (see below) to mice with secondary CE resulted in the complete elimination of the parasite in 95% (19/20) of treated mice, compared to 85% (17/20) of mice treated with liposomal ABZ alone. Apparent toxic effects were reported neither in medicated mice nor in mammalian cell (Neuro2a and HEK293) cultures. In addition, *in vitro* incubation of *E*. *granulosus* protoscoleces with a combination of ABZ and Huaier aqueous extract achieved a moderate (50%) reduction in the number of viable protoscoleces after a prolonged incubation time (12 days) [101].

The pesticide lufenuron (see below) has recently been proposed as an enhancer of the ABZ parasitocidal activity [28]. Although experimentally infected mice with secondary CE separately treated with ABZ or lufenuron did not show a significant decrease in the number and size of cysts compared to untreated controls, the combined administration of both compounds achieved a significant reduction in cyst diameter both per animal (38%) and mice group (28%). These results suggested that a synergistic effect was taking place when these drugs were used in combination, although the actual extent of this finding and its mechanistic explanation should be confirmed in future studies. A synergistic effect was also found when ABZ and metformin, the first-line medication against type 2 diabetes, are combined, both against *E*. *granulosus* protoscoleces and cysts *in vitro* and against *E*. *granulosus* cysts *in vivo* [128,129]. The *in vitro* effect of the combination of BZSO and metformin showed promising results, although 100% mortality of the parasite occurred after prolonged periods of incubation (> 7 days). *In vivo* (mouse model), cyst weight reduction was very high in animals treated with the combined drugs, and higher than the reduction achieved with either metformin or albendazole alone (96% *vs*. 84%). More importantly, reduction in cyst number was also very high in mice treated with the combined drugs, in comparison with animals treated with any of the drugs separately (86% *vs*. 56%).

ABZ-combined treatments not necessarily translate into a significant improvement of the efficacy demonstrated by monotherapeutic approaches, or even elicit inhibitory responses. This seems to be the case of the combined application of ABZ and the synthetic analogue of quinine mefloquine (see below). Thus, whereas monotherapy with ABZ orally administered to *E*. *multilocularis*-infected mice was highly effective in reducing parasite weight, combined application of ABZ under the same regimen with mefloquine orally administered did not increase the treatment efficacy [97]. The fact that intraperitoneal, but not oral, administration of mefloquine was successful in reducing the parasite biomass provided also experimental proof of the relevance of the delivery route in the outcome of the treatment. In another study, the combined treatment of *in vitro*-cultured *E*. *multilocularis* metacestodes with ABZ and the fungicidal agent amphotericin B (S1 Fig, compound 9) exerted an inhibitory effect on vesicle destruction, whereas pre-incubation with ABZ for 24 h and subsequent exposure to amphotericin B exerted a complete destruction of parasite vesicles [19]. Similarly, simultaneous exposure of cultured *E*. *granulosus* protoscoleces to the ABZ derivatives ABZSO and ABZSN for only five min resulted in the killing of 98% of the treated protoscoleces. This treatment efficiency was practically identical to those obtained by ABZSO (98%) and ABZSN (97%) when used in monotherapy [73].

Historically, dual treatment with ABZ and the anthelmintic praziquantel (see below) has been one of the preferred combined therapies against human CE and AE [39,43]. In recent years, new data have become available from *in vivo* animal models and clinical retrospective case series (e.g., in sheep naturally infected with *E*. *granulosus* [108] and in CE patients [130]. However, data on patients are blurry and it is important to highlight that to date there are no published randomized clinical trials comparing treatment with ABZ in monotherapy versus combined therapy with ABZ plus praziquantel, so the potential additive or synergistic effect of these drugs has not been conclusively demonstrated yet [32].

Simultaneous exposure of cultured *E*. *multilocularis* protoscoleces to ABZ and the natural biocide compound thymol (see below) has been reported to obtain a 33% reduction in protoscoleces viability after a prolonged time. This reduction rate was higher than those obtained by ABZ and thymol when individually tested. In addition, treatment with this drug combination was able to induce substantial ultra-structural alteration and severe damage in cultured *E*. *multilocularis* metacestodes, as determined by scanning electron microscopy [131]. The same combination was more effective than ABZ alone against *E*. *multilocularis* established cysts in the mouse model [132].

Apart of ABZ, the only BMZ agents used in combined therapies against *Echinococcus* parasites were FLBZ and OXF (Table 2). Treatment of cultured *E*. *granulosus* protoscoleces with FLBZ plus the anti-parasitic drug ivermectin (see below) evidenced a 96% viability reduction, whereas therapies with FLBZ alone or ivermectin alone were effective in killing ~80% of the protoscoleces. Similarly, *E*. *granulosus* metacestodes cultured *in vitro* and exposed to a dual treatment of FLBZ and ivermectin were damaged faster and to a greater extent than when these drugs were used separately. Vitality assessment was based on the macroscopic appearance of the cysts and the ultra-structural features of the germinal layer [112]. Combination of FLBZ with the synthetic anti-protozoal agent nitazoxanide has been also tested against *E*. *granulosus* protoscoleces and cysts *in vitro* and against cysts in a mouse model [133]. *In vitro*, NTZ is 100% effective after 12 days at 10 μg·mL^-1^, either alone or in combination with FLBZ, while FLBZ alone takes at least 30 days to reach 100% effectivity. In contrast, intragastric administration of FLBZ plus NTZ in infected mice resulted in 65.9% reduction of cyst weight, although FLBZ alone resulted in a higher reduction (80%). These results suggest that the distribution of NTZ *in vivo* could limit its access to the cysts [133].

The therapeutic efficacy of OXF in combination with praziquantel or nitazoxanide (see below) against CE has been evaluated in randomized placebo-controlled trials in naturally infected sheep. Thus, dual oral administration of OXF and nitazoxanide led to a decrease in the number of fertile cysts and an increase in the number of degenerated cysts, achieving a scolicidal activity of 61% for lung cyst and of 100% for liver cysts. These efficacy rates were higher than those found when OXF was used alone (50% and 92%, respectively). No side effects were detected in the treated animals, suggesting that the drug dosages used in this study were safe in sheep. Importantly, the cost of a single dose of OXF for an average weight sheep (32 kg) was estimated at US$ 0.26 [26]. In a subsequent study, oral administration of OXF and praziquantel reduced the parasite biomass by ~72% in both pulmonary and hepatic cysts, although this therapeutic effectivity was not significantly higher than that obtained with OXF alone [108]. Thus, praziquantel activity against CE or AE is still a matter of discussion.

### Synthetic compounds

A wide range of chemically synthetised molecules, including anti-virals, antibiotics, anti-parasites, anti-mycotics, immunomodulatory agents, and inhibitor compounds of key metabolic enzymes or signalling pathways have been evaluated in *in vitr*o and *in vivo* experimental settings in order to assess their potential effect against human CE and AE. However, some of these synthetic compounds have been studied comparatively less thoroughly that their counterparts of the BZD family. Thus, their actual chemotherapeutic efficacy and safety of use remains unclear in many cases. A summary of these molecules and compounds, together with their therapeutic performance is presented in Table 3.

**Table 3:** Characteristics of synthetic compounds for the treatment of human cystic and alveolar echinococcosis based on the published literature.

Compound	Disease	Assay setting			Dosage	Treatment		Efficacy assessment		Toxicity	References
		*In vitro*	*In vivo*	In patients (*n*)		Initiation (p.i.)	Duration	Activity against	Success^a^ (%)		
**ANTIVIRALS AND ANTIBIOTICS**											
Benzoic acid thioureides	AE	Yes	—	—	2–20 μM	N/A	1 week	Cysts	Yes (30-˃50)	N/A	[20]
Cetrimide	CE	Yes	Yes^c^^,^^d^	—	1.5% (cetrimide) + 0.5% (chlorhexidine) (*in vitro*)	N/A	10 min (*in vitro*)	PSC	Yes (93, *in vitro*)	No	[15,73]
					0.1–5% (*in vivo*)^f^		1 min (*in vivo*)		Yes (100, *in vivo*)		
Chlorhexidine gluconate	CE	—	Yes^b^	Yes (30)	0.04% (*in vivo*)^f^	N/S	5 min	PSC	Yes (100, *in vivo*)	N/S	[16,17]
Fluoride	CE	Yes	—	—	20%	N/A	16 h	PSC	Yes (100)	N/A	[140]
Glucose 50%	CE	Yes	—	—	50%	N/A	5 min	PSC	Yes (95)	N/A	[134]
H_2_O_2_	CE	Yes	—	See text	4%	N/A	10 min	PSC	Yes (96)	N/A	[73]
Isoprinosine	AE	Yes	Yes^b^	—	2 mM (i*n vitro*)	N/A (*in vitro*)	48 h (*in vitro*)	PSC, Cysts	Yes (95, *in vitro*)	N/S	[146,147]
					0.5 g·kg^-1^·day^-1^ (*in vivo*)^e^	3 moths (*in vivo*)	2 weeks (*in vivo*)		Yes (42, *in vivo*)		
	CE	—	Yes^b^	—	1–2 g·kg^-1^·day^-1,^^e^	10 months	2 weeks to 2 months	Cysts	Yes (80–90)	N/S	[143]
Monensin	CE	Yes	—	—	10 μM	N/A	3 days	PSC	Yes (100)	N/A	[137]
Nitrite and peroxynitrite	CE	Yes	—	—	80–320 μM	N/A	1–2 days	PSC	Yes (100)	N/A	[138]
Povidone-iodine	CE	Yes	—	See text	10%	N/A	N/S	PSC	Yes (100)	N/A	[90]
Rifampicin	AE	Yes	—	—	10 μg·mL^-1^	N/A	6 weeks	Cysts	No	N/A	[144,145]
	CE	—	Yes	—	9 mg·kg^-1,^^e^	3 months	2 weeks	Cysts	No	N/S	[142]
Saline 20%	CE	Yes	—	See text	20%	N/A	10 min	PSC	Yes (99–100)	N/A	[73,90]
Sodium arsenite	CE	Yes	—	—	20 M	N/A	6 days	PSC	Yes (100)	N/A	[141]
Taurolidine	CE	Yes	Yes^b^	—	5 mg·mL^-1^ (*in vitro*)	N/A (*in vitro*)	1.5 h (*in vitro*)	PSC	Yes (100, *in vitro*)	No	[139]
					400 mg·kg^-1^ (*in vivo*)^g^^,^^h^	0–3 months (*in vivo*)	Single dose (*in vivo*)		Yes (prophylaxis) No (therapy)		
Trimetoprim-sulfametoxazole	AE	Yes	—	—	0.02 mg·mL^-1^	N/A	4 weeks	Cysts	No	N/A	[144]
**ANTIPROTOZOA**											
Artemisinin and synthetic ozonide derivates	CE	Yes	—	—	40 μM	N/A	4 days	PSC	Yes (90)	N/A	[95]
	AE	Yes	Yes^b^	—	20–40 μM (*in vitro*)	N/A (*in vitro*)	4–5 days (*in vitro*)	Cysts	Yes (100, *in vitro*)	Yes	[95,149]
					200 mg·kg^-1^·day^-1^ (*in vivo*)^e^	2 months (*in vivo*	6 weeks (*in vivo*)		Yes (75–100, *in vivo*)		
Mefloquine	AE	Yes	Yes^b^	—	60 μM (*in vitro*)	N/A (*in vitro*)	2–5 days (*in vitro*)	Cysts	Yes (100, *in vitro*)	Yes (see [152])	[97,152]
							8 weeks (*in vivo*)^g^				
					25 mg·kg^-1^·day^-1^ (*in vivo*)^g^	1.5 months (*in vivo*)^e^^. g^	12 weeks (*in vivo*)^e^		Yes (100^g^, 78^e^, *in vivo*)		
					100 mg·kg^-1^ 3 days^-1^ (*in vivo*)^e^						
Mefloquine enantiomer DB1127	AE	Yes	Yes^b^	—	20 μM (*in vitro*)	N/A (*in vitro*)	5 days (*in vitro*)	Cysts	Yes (100, *in vitro*)	Yes	[98,153]
					50 mg·kg^-1^·day^-1^ (*in vivo*)^e^		4 weeks (*in vivo*)^e^		No (*in vivo*)^e^		
					2 mg·kg^-1^·day^-1^ (*in vivo*)^g^	1.5 months (*in vivo*)	6 weeks (*in vivo*)^g^		Yes (70, *in vivo*)^g^		
Miltefosine	AE	Yes	—	—	7.5 μg·mL^-1^	N/A	6 weeks	Cysts	No	N/A	[144,145]
Nitazoxanide	AE	Yes	Yes^b^	Yes (6)	10 μg to 5 mg·mL^-1^ (*in vitro*)	N/A (*in vitro*)	10 days-6 weeks (*in vitro*)	Cysts	Yes (variable, *in vitro* and *in vivo*)	Yes	[5] [6] [22] [145] [150] [154]
					3 mg·day^-1^ (*in vivo*)^e^	0–2 months (*in vivo*)	35 days *(in vivo*)				
					N/S (in patients)	15 months (in patients)	15 months (in patients)		No (in patients)		
	CE	—	Yes^c^	See text	15 mg·kg^-1^·week^-1^ or 30 mg·kg^-1^·day^-1^ (*in vivo*)^e^	N/A	5 days to 5 weeks	Cysts	No	No	[26]
Nitazoxanide derivates RM4807 and RM4841	AE	Yes	—	—	5 mg·mL^-1^	N/A	5 days	Cysts	Yes (N/S)	N/A	[22]
**ANTIHELMINTHIC**											
Clorsulon	AE	Yes	—	—	15 μg·mL^-1^	N/A	20 days	Cysts	No	N/A	[120]
Ivermectin	AE	Yes	—	—	1.75 mg·mL^-1^	N/A	6 weeks	Cysts	No	N/A	[144,145]
	CE	Yes	Yes^b^	—	0.1 mg·mL^-1^ (*in vitro*)	N/A (*in vitro*)	50–72 h (*in vitro*)	PSC, Cysts	Yes (100, *in vitro*)	N/S	[160] [161] [162] [163]
					4 mg·kg^-1^·day^-1^ (*in vivo*)^e^	48 h or 4 months (*in vivo*)	4 months (*in vivo*)		No (*in vivo*)		
Levamisole	CE	Yes	—	—	0.1 mg·mL^-1^	N/A	6 days	PSC	Yes (100)	N/A	[160]
Praziquantel	CE	Yes	—	See text	0.1 mg·mL^-1^	N/A	48 h	PSC	Yes (100)	N/A	[14]
	AE	Yes	Yes^b^	See text	10 μg·mL^-1^ (*in vitro*)	N/A (*in vitro*)	N/S (*in vivo*)	PSC, Cysts	Yes (100, *in vitro*)	N/S	[164,165]
					300 mg·kg^-1^·day^-1^ (*in vivo*)^e^	1 month (*in vivo*)	69 days (*in vivo*)		No (*in vivo*)		
**ANTIARTHROPODA**											
Lufenuron	CE	—	Yes^b^	—	100 mg·kg^-1^·day^-1,^^i^	4 months	4 months	Cysts	No	N/S	[28]
**ANTIMYCOTIC**											
Caspofungin	AE	Yes	—	—	0.07 mg·mL^-1^	N/A	1.5 months	Cysts	No	N/A	[144]
Itraconazole	AE	Yes	—	—	0.7 mg·mL^-1^	N/A	5 weeks	Cysts	No	N/A	[144,145]
**IMMUNOMODULATORS**											
Cyclosporin A	AE	—	Yes^b^	—	40 mg·kg^-1^·day^-1,^^i^	1.5 months	80 days	Cysts	No	N/A	[170]
	CE	Yes	Yes^b^	—	50 μg·mL^-1^ (*in vitro*)	N/A (*in vitro*)	15 days (*in vitro*)	PSC, Cysts	Yes (100, *in vitro*)	N/S	[168,169]
					50 mg·kg^-1^·day^-1^ (*in vivo*)^i^	4.5 months (*in vivo*)	5 days (*in vivo*)		Yes (42, *in vivo*)		
**ANTINEOPLASTIC**											
Bortezomib	AE	Yes	Yes^b^	—	0.5 μM (*in vitro*)	N/A (*in vitro*)	5 days (*in vitro*)	Cysts	Yes (100, *in vitro*)	N/S	[177]
					0.7 mg or 0.5 kg^-1^·week^-1^ (*in vivo*)^g^	6 weeks (*in vivo*)	3 weeks (*in vivo*)		No (*in vivo*)		
Doxorubicin (nanoparticles)	AE	—	Yes^b^	—	5 mg·kg^-1,^^h^	70–80 days	2 doses	Cysts	Yes (60)	N/S	[179]
Genistein and derivates	AE, CE	Yes	—	—	10 μg·mL^-1^	N/A	4 to 14 days	PSC, Cysts	Yes (60–100 for PSC, N/S for cysts *in vitro*)	N/A	[173]
Imatinib	AE	Yes	—	—	50 μM	N/A	1–3 weeks	Cells, PSC, Cysts	Yes (100)	N/A	[174]
5-fluorouracil, Paclitaxel	CE	Yes	—	—	10 μg·mL^-1^	N/A	7–72 days	Cells, PSC, Cysts	Yes (100, for 5-fluorouracil)	N/A	[175]
Methotrexate, Navelbine, Vincristine	AE	Yes	—	—	2.4–60 nM	N/A	5 days	Cysts	No	N/A	[171]
2-Methoxyestradiol	CE	Yes	—	—	10 μM	N/A	1 week	Cysts	Yes (100)	N/A	[172]
	AE	Yes	Yes^b^	—	10 μM (*in vitro*)	N/A (*in vitro*)	1 week (*in vitro*)	Cysts	Yes (100, *in vitro*)	N/A	[172]
					200 mg·kg^-1^·day^-1^ (*in vivo*)^e^	2 months (*in vivo*)	6 weeks (*in vivo*)		No (*in vivo*)		
Mitomycin C	AE	—	Yes^b^	—	0.1 mg·week^-1,^^g^	1 day or 1 month	3 weeks	Cysts	Yes (76)	N/A	[180]
η(6)-Areneruthenium (II) phosphite	AE	Yes	—	—	20 μM	N/A	5 days	Cysts	Yes (100)	N/A	[176]
Tamoxifen	CE	Yes	Yes^b^	—	20 μM (*in vitro*)	N/A (*in vitro*)	2–7 days (*in vitro*)	PSC, Cysts	Yes (100, *in vitro*	N/S	[25]
					20–200 mg·kg^-1^·day^-1^ (*in vivo*)^e^	3–6 months (*in vivo*)	32–80 days (*in vivo*)		Yes (61–83, *in vivo*)		
**GENOME-BASED**											
Kinase inhibitor BI2536	AE	Yes	—	—	50 nM	N/A	2–3 weeks	Cells, Cysts	Yes (100 for cysts; 98 for cells)	N/A	[182]
Kinase inhibitors ML3403 and SB202190	AE	Yes	—	—	5 μM	N/A	4 days (cysts), 4 weeks (cells)	Cells, Cysts	Yes (100 for cells, 50 for cysts)	N/A	[183]
Kinase inhibitor SB202190	CE	Yes	—	—	80 μM	N/A	1 day	PSC	Yes (70)	N/A	[184]
**OTHERS**											
Chenodeoxycholic acid	CE	Yes	—	—	2 M	N/A	10 days	PSC	Yes (100)	N/A	[188]
Selenium nanoparticles	CE	Yes	—	—	500 μg·mL^-1^	N/A	10 min	PSC	Yes (100)	N/A	[186]

N/A: Not applicable; N/S: Not specified; PSC: Protoscoleces.

^a^ Defined as a significant reduction of the parasite burden or the viability of the any form of the larval stage of the parasite, including protoscoleces or cysts.

^b^ Experimental infection in mice.

^c^ Natural infection in sheep.

^d^ Natural infection in cattle.

^e^ Oral or intra-gastric administration.

^f^ Intracystic injection administration.

^g^ Intraperitoneal administration.

^h^ Intravenous administration.

^i^ Subcutaneous administration.

Following, detailed information is given on the different compounds, their mode of action (when available), and effectivity against *E*. *granulosus* and *E*. *multilocularis in vitro* and *in vivo* at the different dosages and administration regimes assayed by different authors. While information on the use of BMZ against AE and CE has been compiled only from 2008, drugs and compounds different from BMZ are presented historically, allowing the reader to identify most of the agents that have been assayed to seek for their potential use in the treatment of human AE and CE.

#### Anti-virals and antibiotics

Fifteen different anti-viral and antibiotic compounds have been tested against CE and/or AE (Table 3). The majority of them have been specifically assessed for their scolicidal activity and potential use to avoid recurrence in CE, either intra-operatively in the peri-cystic area or directly injected into the cysts during surgical intervention or percutaneous management.

Currently, 20% saline in contact with the germinative membrane of cysts (intra-cyst) for at least 15 min is recommended to avoid recurrences in CE [3]. Nevertheless, 20% saline can induce cholangitis when the cyst is communicated with bile ducts. Thus, alternative compounds with scolicidal activity have been tested by different authors, the majority of them presenting a generic mechanism of action directed towards biological membranes.

Cetrimide (one of whose active components is cetrimonium bromide; S1 Fig, compound 10), 50% glucose, H_2_O_2_, monensin (S1 Fig, compound 11), nitrite, and peroxynitrite, povidone-iodine (S1 Fig, compound 12), taurolidine (S1 Fig, compound 13), sodium arsenite, and fluoride have been tested on *E*. *granulosus* protoscoleces *in vitro*. The effect of 50% glucose on protoscoleces was promising [134], although the adverse effects of high concentrations of glucose on host cells should be further assessed.

H_2_O_2_ and povidone-iodine have shown scolicidal activity *in vitro* at 4% and 10%, respectively [73,90], but have been more frequently used peri-operatively covering the peri-cystic interventional area with patches soaked with diluted solutions of one of these compounds. Due to their toxicity, the intra-cyst use of H_2_O_2_ and povidone-iodine has been performed in very few cases [135,136] and its general use for intra-operative interventions cannot be recommended.

Monensin, nitric oxid derivates, taurolidine, sodium arsenite, and fluoride have shown scolicidal effect after prolonged *in vitro* incubation times (≥ 12 h), but the activity of these compounds at the time periods (min) usually employed for scolicidal (intra-cyst) treatment have not been reported [137–141]. Taurolidine has been also tested *in vivo* immediately after intra-peritoneal inoculation of mice with protoscoleces, showing 100% scolicidal activity after a single intravenous or intra-peritoneal dose of 400 mg·kg^-1^ [139]. Interestingly, taurolidine showed potential scolicidal activity *in vivo* at the same dose regimen in mice treated after 3 months post-infection, period in which recovered cysts were all sterile, without adverse effects. Nevertheless, taurolidine did not show activity against the metacestode besides its scolicidal effect [139].

Additionally, cetrimide and chlorhexidine gluconate (S1 Fig, compound 14) have been used as scolicidals after intracyst injection [17,73]. Low concentration (0.04% to 0.1%) and short exposure times (1 to 5 min) for both compounds were ~100% effective against protoscoleces inside the treated cysts. Chlorhexidine gluconate alone has been also used in patients, showing 100% activity against protoscoleces after intra-cyst injection of a 0.04% solution for 5 min, with no side effects reported [16].

The anti-viral isoprinosine (S1 Fig, compound 15) and the antibiotic rifampicin (S1 Fig, compound 16) have been also tested against CE. Both were given orally to mice after 3.3 and 10 months post-infection, respectively. Rifampicin did not show any effect against an established CE infection [142], while isoprinosine at different doses and treatment duration (see Table 3) resulted in a 80–90% decrease of the mean cyst weight and microscopical alterations of the cyst germinal membrane and protoscoleces in treated animals compared with non-treated mice [143]. Similar results were found when the two compounds were used against *E*. *multilocularis*. Rifampicin, an inhibitor of the DNA-dependent RNA polymerase enzyme in bacterias, did not show efficacy against AE cysts *in vitro* [144,145], and isoprinosine was only partially effective against both *E*. *multilocularis* protoscoleces *in vitro* and metacestode vesicles in mice with secondary AE [146,147]. The widely used trimethoprim-sulfametoxazole (S1 Fig, compound 17) antibiotics, also inhibitors of bacterial enzymes responsible for the synthesis of folic acid, did not show activity against AE cysts *in vitro* [144]. Importantly, the mechanism of action of isoprinosine is also related with the immune system, and it has been shown to restore impaired cell-mediated immune response to its baseline level, in addition to enhancing humoral immune responses [148].

Fifty members of a novel class of anti-microbial compounds, 2-(4-R-phenoxymethyl) benzoic acid (S1 Fig, compound 18) thioureides, were synthesized and characterized with respect to their activity against AE cysts *in vitro* [20]. Two compounds (14 and 49) showed the strongest cysticidal effect, resulting in the damage of 30% to more than 50% of cysts at concentrations of 2 to 20 μM after incubation for 1 week, without exerting toxic effects in host cells [20]. The mode of action of thioureides in eucaryotes is unknown, so far, but due to their activity *in vitro* against AE cysts at non-toxic concentrations, their testing *in vivo* should be performed.

#### Anti-protozoa

Several drugs usually used against protozoan parasites have been tested mainly against *E*. *multilocularis* vesicles both *in vitro* and *in vivo* (Table 3). Artemisinin (S1 Fig, compound 19) and its synthetic ozonide derivatives (S1 Fig, compound 20), mefloquine (S1 Fig, compound 21) and its enantiomers, and nitazoxanide (S1 Fig, compound 22) and its derivatives have shown high activity against AE cysts *in vitro* [5,6,22,95,97,145,149,150]. On the contrary, miltefosine (S1 Fig, compound 23), which has been postulated to reduce ATP and GTP synthesis, showed to be ineffective against AE cysts *in vitro* at concentrations similar to those used for the above-mentioned compounds [144,145]. Only three of these drugs (artemisinin, mefloquine plus enantiomers, and nitazoxanide) have been further tested in the *in vivo* AE mouse model.

The semi-synthetic derivative of artemisinin artesunate (S1 Fig, compound 24), potentially activating a cascade of reactions leading to the generation of reactive oxygen radicals which damages parasites, did not affect alone parasite growth in mice orally treated at 2 months post-infection, but improved the effects of ABZ when combined [95], probably due to the enhanced entry of artesunate inside ABZ-damaged cysts. Although toxic *in vitro* [95,149] and in several *in vivo* models, artemisinin and its derivatives are rapidly eliminated after oral intake, representing a relatively safe route of administration in patients [151]. Artemisinin has shown to be also effective against *E*. *granulosus* protoscoleces *in vitro* after 4 days of incubation [94]. Thus, it could be worth assessing the scolicidal activity of this drug in the *in vivo* murine CE model.

Mefloquine has been tested *in vitro* and showed a dose-dependent cysticidal activity of 100% at 24 μM for 10 days, as determined by murine bioassays [97]. *In vivo*, oral dosage and intraperitoneal administration of mefloquine in mice against AE, resulted in a decrease in parasite weight–parasitostatic effect–comparable to that obtained after oral ABZ treatment, without increase in treatment efficacy when both drugs were given together [97,152]. The *in vivo* dose of mefloquine used in these studies was 20-fold lower than the LD_50_ in rats (880 mg·kg^-1^) when administered orally (http://www.drugbank.ca/drugs/DB00358), but potential toxicity of this drug in the intraperitoneal route should be further assessed if it should be recommended as alternative to ABZ. Similar results were obtained *in vitro* and *in vivo* for the DB1127 (S1 Fig, compound 25) enantiomer of mefloquine [98,153]. The parasitostatic effects of mefloquine *in vivo* could be attributed to its binding to ferritin and cystatin of the parasite [152].

Nitazoxanide has been the most extensively studied compound in this group of drugs. As previously mentioned, nitazoxanide has shown high activity against AE cysts both *in vitro* and *in vivo* given orally, with variable effectivity depending on the exposure time and the dose [5]. Due to those promising results, nitazoxanide has been used for the treatment of AE patients. Unfortunately, available data on patients’ therapies are limited to a comparatively low number of cases under heterogeneous regime treatments and clinical scenarios. For instance, in six patients treated with nitazoxanide for a median of 15 months, no improvement was observed and side effects were detected in some of the treated cases [154]. Similarly, in a single patient, monotherapy with 1 g nitazoxanide for 15 months did not stop parasite growth [155].

Nitazoxanide has also reached clinical use, in combination with ABZ against CE in patients, but again studies are on a very limited number of patients with different clinical and management conditions. Contradictory results have been reported using similar treatment regimens for bone CE patients (stable versus progressive disease after treatment) [156,157]. Similarly, contradicting results were also obtained when nitazoxanide in combination with ABZ and praziquantel were used for the treatment of five CE patients, although conclusions are difficult to drawn due to the heterogeneity in the management of each patient [158]. Nitazoxanide has also been used for the treatment of CE *in vivo* on naturally infected sheep, showing no effects on cysts from treated animals [26]. Together, these results discourage the further use of nitazoxanide against *Echinococcus*.

#### Anthelmintic

Four different anti-helmintic drugs have been assayed against *E*. *granulosus* and *E*. *multilocularis in vitro* (Table 3). Clorsulon (S1 Fig, compound 26), an inhibitor of phosphoglycerate mutase and kinase with a selective inhibition of glucose utilization, has shown activity against flukes [159]. Despite the fact that both enzymes are known to be metabolically expressed in *E*. *multilocularis* [31], when used on *E*. *multilocularis* metacestodes *in vitro* clorsulon did not show activity against the parasite [120]. This lack of activity could be attributed to sequence differences in the two enzymes between fasciolids and *Echinococcus*.

Ivermectin (S1 Fig, compound 27) is used against nematodes and increases Cl^-^ permeability in the parasite’s muscle cells, resulting in parasite paralysis [159]. A paralyzing effect followed by irreversible tissue vacuolization and increased HSP60 expression was shown after *in vitro* exposure of *E*. *granulosus* protoscoleces to both ivermectin and levamisole (S1 Fig, compound 28) (an agonist of nicotinic receptors producing spastic paralysis in worms), although after prolonged (˃ 48 h) treatment periods [160,161]. Direct intra-cyst injection of ivermect at laparotomy in a rodent model also resulted in scolicidal activity, as demonstrated by the damaged germinative membranes found in injected cysts [162]. In contrast, ivermectin showed no *in vivo* effect in the CE secondary infection mouse model when given orally either at 48 hours or at 4 months post-infection, although it showed synergistic effects when given in combination with ABZ [163]. Ivermectin-induced damage of the germinal membrane after direct intra-cyst injection but lack of effect when administered orally could be due to the limited access of the drug into the cyst in *in vivo* conditions, rather than to the actual effectivity of the drug against the cyst structures. Similarly, ivermectin has also been tested against *E*. *multilocularis* vesicles *in vitro*, showing no effect at high concentration and prolonged exposure times [144,145].

Praziquantel (S1 Fig, compound 29) causes rapid muscle contraction in schistosomes due to the induction of alterations in Ca^2+^ permeability of parasites’ cells. Praziquantel has been tested *in vitro* and *in vivo* against both CE and AE. This compound was active against *E*. *granulosus* protoscoleces and *E*. *multilocularis* metacestodes *in vitro* [14,164]. Regarding its activity against protosocoleces, there is some clinical evidence supporting the role of praziquantel in combination with ABZ in CE patients to reduce the risk of disease recurrence, although this therapeutic regimen should be further evaluated for efficacy and safety in humans [3]. Against the cyst stage, few data are available, either from prospective assays in naturally infected sheep [108] or from retrospective studies in patients [32,130], for the recommendation of its use, either alone or in combination with other drugs, in prolonged drug treatment for chronic CE. For AE, experimental data showed that praziquantel enhances cyst growth in jirds [165] and thus cannot be recommended for the treatment of AE patients. The voltage-gated calcium channel subunit beta, the proposed target of praziquantel in schistosomes, is not expressed in *Echinococcus* cysts [31], providing a putative explanation for its apparent lack of efficacy *in vivo* against CE or AE cysts, and discouraging further testing in patients.

#### Anti-arthropoda

Only a single anti-arthopoda drug, the insect growth inhibitor lufenuron (S1 Fig, compound 30), has been tested against CE in the *in vivo* mouse model (Table 3). After secondary infection of mice, the subcutaneous administration of lufenuron at 4 months post-infection was demonstrated to be ineffective against the established parasite [28]. However, luferunon enhanced the activity of ABZ when administered together, and its effect is attributed to the interference with the laminated layer cyst development [28]. One of the main problems associated with the efficacy of drugs against CE established cysts is to reach enough drug concentration inside the cyst to be active against the parasite. The mechanical barrier represented by the laminated layer of the metacestode, and the low solubility of most of the drugs used or assayed against CE and AE, hinders the passive drug entry into the cyst. Importantly, luferunon is the only known compound that could interfere with the formation of the laminated layer in established CE cysts, thus potentially facilitating the access of other drugs to the live parasite tissue inside the cyst. Further studies on the mechanisms by which luferunon interferes with the laminated layer formation could lead to the formulation of compounds to be used in combination with BMZ in the treatment of CE patients.

#### Anti-micotics

The activity of the anti-fungal drugs caspofungin (S1 Fig, compound 31) and itraconazole (S1 Fig, compound 32) has been tested against *E*. *multilocularis* vesicles *in vitro* (Table 3). Both are selective inhibitors of fungal growth by interfering with the fungal cell wall synthesis. Caspofungin showed to be ineffective, while itraconazole provoked cyst disintegration effects, but regrowth of the parasite was shown at seven days after the discontinuation of incubation with the drug. Similar effects were found when cysts were incubated with ABZ [144,145]. Whether the reversible effects of drugs like itraconazole and ABZ on AE cysts could be due to the selective action on specific cells of *E*. *multilocularis* and not on cells particularly important for parasite survival and re-growth after drug treatment discontinuation should be further investigated. Additionally, amphotericin B has been used as salvage treatment for AE patients with intolerance or resistance to benzimidazoles [166]. Although amphotericin B was not parasitocidal, intravenous doses of 0.5 mg·kg^-1^ of body weight three times per week effectively halted disease progression in three patients. Because amphotericin B is commonly used to inhibit fungal growth in culture media, its parasitostatic properties may have inadverted, detrimental effects in *in vitro* experimental settings involving *E*. *multilocularis* vesicles.

#### Immunomodulators

Cyclosporine A (S1 Fig, compound 33) is an immunosuppressant that specifically and reversibly blocks the transcription of cytokine genes in activated T cells [167]. Cyclosporine has been tested against AE and CE cysts in the mouse model and against *E*. *granulosus* protoscoleces *in vitro* (Table 3). A prolonged exposure time was needed for scolicidal activity *in vitro* [168], precluding its use by direct injection into cysts for a rapid scolicidal activity, but showing its potential use for pre- and post-treatment in CE patients subjected to surgery or aspiration techniques. Interestingly, cyclosporine has shown activity against protoscoleces and cysts in the CE mouse model. When administered subcutaneously 2 days prior to infection, the total number of cysts was around 18-fold lower than that found in non-treated, infected mice [169]. The effect was less pronounced when treatment started at 4.5 months post-infection, resulting in a ~50% decrease in cyst weight in treated animals compared with non-treated mice [169].

When cyclosporine has been used at a similar regime treatment against AE in mice, the immunosuppressive effects resulted in the enhanced growth of the parasite in treated animals [170]. Cyclosporine is used in AE patients after liver transplantation, and the *in vivo* results obtained with this drug shows the need of a careful follow-up of recurrences in this group of patients.

#### Anti-neoplastics

Numerous anti-neoplastic drugs have been tested for activity against *E*. *granulosus* and *E*. *multilocularis* (Table 3). In general, it should be mentioned that these compounds have well defined targets as anti-cancer compounds in mammalian cells, although their potential targets in *Echinococcus* have usually not been investigated, thus their effects cannot be attributed to the presence of target homologues in the parasite. Additionally, and similar than to other drugs assayed against *Echinococcus*, dose of anti-cancer drugs reported by some authors are too high to be safely used in patients. Two inhibitors of mitosis due to their interaction with tubulin named navelbine (S1 Fig, compound 34) and vincristine (S1 Fig, compound 35), in addition to methotrexate (S1 Fig, compound 36), an inhibitor of folic acid reductase leading to inhibition of DNA synthesis, have been shown to be ineffective against *E*. *multilocularis* vesicles *in vitro* [171], either due to low affinity of these compounds against the specifically targeted molecules in *E*. *multilocularis* or to the scanty access of these drugs inside the cysts. Similarly, 2-methoxyestradiol (S1 Fig, compound 37), an angiogenesis inhibitor, showed to be ineffective against both CE and AE cysts *in vitro* and against AE in the mouse model [172]. Treatment of AE cysts *in vitro* with this drug showed profound alterations of the germinative layer of the parasite as seen in transmission electron microscopy, but drug-induced damage was not enough to avoid parasite growth after subsequent injection of the AE-treated material into mice [172]. Although this effect could be attributed to the need of prolonged treatments to achieve full effectiveness of defined compounds, this study provided experimental evidence showing that, in order to be conclusively considered as a cysticidal agent, any drug assessed *in vitro* has to achieve the complete inactivation of the cyst. The *in vivo* treatment of AE infected mice with a combination of 2-methoxyestradiol and ABZ at 2 months post infection did not show higher reduction in cyst weight in treated animals than in animals treated only with ABZ [172]. Thus, this study is also a good example to illustrate that, to some extent, the laminar layer surrounding the metacestode actively contributes with regard to protectivity against drugs.

A number of anti-neoplastic drugs have shown *in vitro* activity against stem cell, protoscoleces and cysts/vesicles of *E*. *granulosus* and *E*. *multilocularis*. Genistein (S1 Fig, compound 38) and derivatives, imatinib (S1 Fig, compound 39), 5-fluorouracil (S1 Fig, compound 40), paclitaxel (S1 Fig, compound 41) and tamoxifen (S1 Fig, compound 42) are scolicidal *in vitro*, all them after prolonged (˃ 24 hours) exposure time, and could be pre- and post-treatment candidate drugs to prevent recurrences in CE [25,173–175]. All those drugs, together with areneruthenium complexes (S1 Fig, compound 43) [176] and bortezomib [177,178], showed also activity against CE or AE cysts *in vitro* (Table 3), but only two–bortezomib and tamoxifen–were further tested *in vivo* [25,177]. Tamoxifen showed 100% activity against CE protoscoleces *in vitro*, although this rate was reduced to 40% (6 out of 10 treated mice developed cysts) when the treatment was given orally to mice at infection. When used at 3 or 6 months post infection, treatment resulted in parasitostatic effects (reduction in cyst weight), which were more pronounced in early (3 months) than in late (6 months) post infection regimens [25]. Tamoxifen binds to estrogen receptors (ER), and if *E*. *granulosus* is sensitive to estrogens the candidate receptor might be the *E*. *granulosus* ER described in this study [25]. The proposed short-term, low-dose therapy assessed by the authors could be a novel alternative approach for human CE treatment. In contrast, although bortezomib treatment resulted in a clear effect on the morphological structure of *E*. *multilocularis* lesions, both *in vitro* and *in vivo*, in terms of parasite weight no significant reduction compared to untreated animals was found, thus giving evidence of potentially low effectivity of this compound against AE [177].

Additionally, doxorubicin (S1 Fig, compound 44) and mitomicyn C (S1 Fig, compound 45) have shown some effect on AE cysts in the rodent model. Doxorubicin, bound to a colloidal biodegradable drug carrier, was tested in jirds infected by intrahepatic injection of AE metacestodes [179]. Two intravenous doses at 70 and 80 days post-infection resulted in the absence of liver parasite lesions in 60% of treated animals, compared with the non-treated control group. Surprisingly, when one additional dose was given to the animals at day 90 post-infection, animals without liver lesions were only 10%. When parasite burden in the peritoneum was assessed, no differences were found between treated and non-treated animals [179]. These results, together with the lower effect achieved by the administration regimen of three doses instead of two doses, question the real effectiveness of this treatment against AE in this experimental model.

Mitomicyn C was effective against AE in jirds intraperitoneally administered either at 1 day or at 1 month post-infection, reducing ~70% the parasite weight in treated animals [180]. Nevertheless, the three doses of 0.1 mg each weekly described by the author resulted in the death of 25% of the treated animals. The same author tried lower doses with the same anti-parasitic effects and no adverse reactions, but only administered at 1 day post-infection [180].

Many anti-cancer drugs primarily target cells in mitosis. In this sense, three anti-neoplastic drugs have been tested for activity against stem cells from both *E*. *granulosus* (5-fluorouracil and paclitaxel; [175]) and *E*. *multilocularis* metacestodes (imanitib [174]). *E*. *granulosus* cells were incubated for 7 days with 10, 5, and 1 μg·mL^-1^ of 5- fluorouracil or paclitaxel, and cell viability was assessed by trypan blue exclusion and scanning electron microscopy. At 10 μg·mL^-1^, both drugs inhibited cell proliferation and reduced the original cell number, whereas the remaining cells were contracted and blebbing [175]. On the other hand, *E*. *multilocularis* larvae have been shown to express potential targets for imanitib [174]. This drug had deleterious effects on both cysts and protoscoleces of the parasite *in vitro*. Additionally, exposure of parasite stem cells to imanitib (at 25 or 50 μM) resulted in the complete abolition of new vesicle formation [174]. *In vivo* studies should be undertaken to further investigate whether imanitib treatment could have a beneficial effect in the treatment of AE patients.

#### Genome-based targets

Recently, a set of 250–300 kinases has been identified in the *Echinococcus* genome [31,181]. Kinases bind both their specific substrate and ATP, thus they can be inhibited by small molecule compounds [181]. Due to their importance in malignant transformation, research already brought up several inhibitory compounds currently in use to treat various forms of cancer [181]. Not surprisingly, several anti-neoplastic drugs tested against *Echinococcus* are kinase inhibitors (e.g., imatinib and pyridinylimidazoles; Table 3). These and other anti-neoplastic drugs with this mode of action have been leading compounds for the design of related small molecules that show therapeutic effects against the parasite. These include the kinase inhibitors BI2536 (S1 Fig, compound 46) [182], ML3403 (S1 Fig, compound 47) and SB202190 (S1 Fig, compound 48) [183,184] (see Table 3). The three of them have been tested *in vitro* against stem cells and metacestodes of *E*. *multilocularis*, and the inhibitor SB202190 has been also assessed *in vitro* against *E*. *granulosus* protoscoleces [184].

BI2536, ML3403, and SB202190 have shown 98–100% activity against *E*. *multilocularis* stem cells at 50 nM and 5 μM, being necessary prolonged incubation times (from 2 to 4 weeks) to achieve full activity with lower compound concentrations [182,183]. Similarly, long incubation times (˃1 week) seem to be also required to exert their activity against whole cysts. In this regard, incubation for 4 days resulted in 50% cyst destruction [183], while 100% activity was reached only after 3 weeks of incubation [182]. The kinase inhibitor SB202190 showed a sub-optimal activity (70%) when tested against *E*. *granulosus* protoscoleces at a much higher concentration (80 μM) than that showing activity against *E*. *multilocularis* stem cells and vesicles [184] (Table 3), although the incubation time was shorter (1 day) than the exposure time used against *E*. *multilocularis*. Whether these differences were due to the parasite species or to other factors should be further investigated.

#### Others

Selenium is a micronutrient with anti-oxidant activity that has been used incorporated in nanoparticles to inhibit bacterial growth [185]. Biogenic selenium nanoparticles have been tested against *E*. *granulosus* protoscoleces *in vitro*, showing 100% activity after 10 min incubation of the parasite with 500 μg·mL^-1^ of this compound [186]. This activity was comparable to that of other scolicidal compounds that can be used intra-cyst, making selenium incorporated in nanoparticles a potentially suitable scolicidal candidate. In this sense, nanoparticles preparation and sterilization described by the authors are laborious and time-consuming, but the use of commercially available and homogeneous preparations of nanoparticles could facilitate its use.

Chenodeoxycholic acid (CDCA) is a bile acid commonly used for the treatment of gallstones. Its use as a protoscolicidal agent is justified after the report of induction of apoptosis by bile acids in different cell types, including cancer cells [187]. As such, CDCA has been used *in vitro* against *E*. *granulosus* protoscoleces, and although 100% effective, its effects are seen only after prolonged incubation time (10 days) at high concentration (2 to 3 mol·L^-1^) [188], limiting its use as a protoscolicidal intra-operative agent.

### Natural compounds

In the last few years, a growing number of plant- and fungus-derived products have been tested against CE and AE, seeking for alternative natural compounds for the effective treatment of both diseases. Nevertheless, the vast majority of these molecules have been exclusively assayed *in vitro* against *E*. *granulosus* protoscoleces, and only three of them have been evaluated for its activity in the murine *in vivo* model (Table 4). In general, these compounds are naturally occurring biocides with low toxicity, acting synergistically with synthetic compounds against pathogen resistant strains, and as such they can be used as general purpose disinfectants [189]. Although some of them have shown effects potentially similar to ABZ, none of them have been tested, alone or in combination, in CE or AE patients.

**Table 4:** Characteristics of natural compounds for the treatment of human cystic and alveolar echinococcosis based on the published literature.

Compound	Disease	Assay setting			Dosage	Treatment duration		Efficacy assessment		Toxicity	References
		*In vitro*	*In vivo*	In patients (*n*)		Initiation (p.i.)	Duration	Tested against	Success^a^ (%)		
		**THYMOL, MENTHOL AND PLANT EXTRACTS RICH IN THYMOL**									
Thymol	CE	Yes	Yes^b^	—	50–200 μg·mL^-1^ (PCS and cysts *in vitro*)	N/A (*in vitro*)	1 min to 2 days (PSC and Cysts); 7 days (Cells) (*in vitro*)	PSC, Cysts, Cells	Yes (100 for PSC and Cysts; 63% for Cells, *in vitro*)	No	[142] [190] [191] [193]
					5 μg·mL^-1^ (cells *in vitro*)						
					64 mg·kg^-1^·day^-1^ (*in vivo*)^c^	3.5 months (*in vivo*)	2 weeks (*in vivo*)		No (*in vivo*)		
	AE	Yes	—	—	10 μg·mL^-1^	N/A	10–36 days	PSC, Cysts	Yes (48 for PSC, NA for cysts)	N/A	[131]
Menthol	CE	Yes	—	—	50 μg·mL^-1^	N/A	5 days	PSC	Yes (100)	N/A	[190]
*Trachyspermum ammi L*. fruit essential oil	CE	Yes	—	—	10 mg·mL^-1^	N/A	10 min	PSC	Yes (100)	N/A	[192]
*Zataria multiflora* water or methanol extracts	CE	—	Yes^b^	—	20–40 mL·l^-1,^^d^	0–8 months	3–8 months	PSC, Cysts	Yes (100 in prophylaxis and 93 in therapy)	No	[88,89]
*Thymus vulgaris* and *Origanum vulgare* essential oils	CE	Yes	Yes^b^	—	10 μg·mL^-1^ (*in vitro*)	4 months	5 days to 2 months (*in vitro*)	PSC, Cysts	Yes (60–70 for PSC, N/S for cysts *in vitro*)	N/A	[175] [194]
					40 mg kg^-1^·day^-1^ (*in vivo*)^d^		20 days (*in vivo*)		Yes (72.3 *in vivo*)		
*Salvia officinalis* and *Thymus vulgaris* ethanol extracts	CE	Yes	—	—	500 μg·mL^-1^	N/A	6–7 days	PSC	Yes (100)	N/A	[190]
*Mentha* spp. essential oil	CE	Yes	—	—	10 μg·mL^-1^	N/A	1–18 days	PSC, Cysts, Cells	Yes (50–100 for PSC; NA for cysts; 77% for cells)	N/A	[195] [193]
*Rosmarinus officinalis* essential oil	CE	Yes	—	—	10 μg·mL^-1^	N/A	7 days	Cells	Yes (82%)	N/A	[193]
**PLANT AND FUNGUS EXTRACTS**											
*Allium sativum* methanol or chloroform extract	CE	Yes	—	—	50–200 mg·mL^-1^	N/A	10–180 min	PSC	Yes (98–100)	N/A	[196–198]
*Berberis vulgaris* aequous extract	CE	Yes	—	—	4 mg·mL^-1^	N/A	5 min	PSC	Yes (100)	N/A	[201]
*Cnidium monnieri* osthole	CE	Yes	—	—	120 M	N/A	3 days	PSC	Yes (100)	N/A	[210]
	AE	—	Yes^b^	—	100 mg·kg^-1^·day^-1^	3.5 months	6 weeks	Cysts	Yes (50)	No	
*Corylus* spp. and *Curcurbia* spp. hydroalcoholic extracts	CE	Yes	—	—	50 mg·mL^-1^	N/A	2 h	PSC	Yes (10)	N/A	[197]
*Curcuma longa* ethanol extract	CE	Yes	—	—	50 mg·mL^-1^	N/A	30 min	PSC	Yes (93)	N/A	[206]
*Mallotus philippinensis* fruit methanol extract	CE	Yes	—	—	20 mg·mL^-1^	N/A	2 h	PSC	Yes (99)	N/A	[199]
*Nigella sativa* seed essential oil	CE	Yes	—	—	10 mg·mL^-1^	N/A	10 min	PSC	Yes (100)	N/A	[208]
*Olea europaea* leaves aequous extract	CE	Yes	—	—	0.1%	N/A	2 h	PSC	Yes (90)	N/A	[203]
*Penicillum* extracted chitosan	CE	Yes	—	—	200–400 μg·mL^-1^	N/A	3 h	PSC	Yes (100)	N/A	[212,213]
*Penicillium aculeatum* in silver particles	CE	Yes	—	—	0.15 mg·mL^-1^	N/A	2 h	PSC	Yes (90)	N/A	[214]
*Pestalotiopsis* spp. ethyl acetate extract	CE	Yes	—	—	20 mg·mL^-1^	N/A	30 min	PSC	Yes (100)	N/A	[202]
*Pistacia atlantica* fruit methanol extract	CE	Yes	—	—	50 mg·mL^-1^	N/A	10 min	PSC	Yes (100)	No	[209]
*Punica granatum* peel aequous extract	CE	Yes	Yes^b^	—	16 mg·mL^-1^ (*in vitro*)	2 days	48 h (*in vitro*)	PSC, Cysts	Yes (100, *in vitro*)	N/A	[211]
					650 mg·kg^-1^·day^-1^ (*in vivo*)^d^		2 months (*in vivo*)		Yes (63, *in vivo*)		
*Salvadora persica* root ethanol extract	CE	Yes	—	—	50 mg·mL^-1^	N/A	20 min	PSC	Yes (100)	No	[204]
*Sambucus ebulus* fruit methanol extract	CE	Yes	—	—	100 mg·mL^-1^	N/A	2 h	PSC	Yes (99)	N/A	[200]
*Satureja khuzestanica* leaves hydroalcoholic extract	CE	Yes	—	—	0.1%	N/A	30 min	PSC	Yes (100)	N/A	[203]
*Trametes robiniophila murr* aequous extract	CE	Yes	Yes^b^	—	2 mg·mL^-1^ (*in vitro*)	N/A (*in vitro*)	12 days (*in vitro*)	PSC, Cysts	Yes (20, *in vitro*)	No	[101]
					15 g·kg^-1^·week^-1^ (*in vivo*)^d^	1.5 months (*in vivo*)	4 months (*in vivo*)		Yes (75, *in vivo*)		
*Zingiber officinale* ethanol extract	CE	Yes	—	—	50–100 mg·mL^-1^ (PSC-Cysts)	N/A	10 min (PSC, 72 h (Cysts)	PSC, Cysts	Yes (100)	N/A	[206]

N/A: Not applicable; N/S: Not specified; PSC: Protoscoleces.

^a^ Defined as a significant reduction of the parasite burden or the viability of the any form of the larval stage of the parasite, including protoscoleces or cysts.

^b^ Experimental infection in mice.

^c^ Intramuscular administration.

^d^ Oral or intragastric administration.

#### Thymol, menthol and plant extracts rich in thymol and menthol

Thymol (S1 Fig, compound 49), a natural monoterpene phenol, and plant extracts rich in this compound, have shown strong antiseptic properties. Commercial thymol and in-house made (essential oil, water, methanol or ethanol) extracts showed dose- and time-dependent activities when tested *in vitro* against *E*. *granulosus* (Table 4). Thymol at 50 to 200 μg·mL^-1^ applied *in vitro* during seconds to few minutes resulted in the death of 100% of treated protoscoleces, inducing ultrastructural damage to 100% of *in vitro* and *in vivo* obtained cysts [190,191]. In contrast, *in vitro* treatment of protoscoleces and vesicles of *E*. *multilocularis* with 10 μg·mL^-1^ thymol only resulted in partial loss of viability even at prolonged (10 or more days) incubation times [131]. The different effects obtained with thymol against both parasite species could be due to the need of a minimum concentration of the compound (≥50 μg·mL^-1^) to exert its full parasitocidal activity. For plant extracts rich in thymol such as *Trachyspermum ammi* and *Origanum vulgare* essential oils, and *Salvia officinalis* and *Thymus vulgaris* ethanol extracts, 100% scolicidal activity have been achieved against *E*. *granulosus* at different concentrations and incubation times [175,190,192] (Table 4). On the contrary, the essential oil of *Thymus vulgaris* showed only 70% scolicidal activity [175]. In-house made extracts could contain variable amounts of the active compound depending on the extraction procedure. Therefore, interpretation of efficacy results could be greatly facilitated if commercial and standarised extracts are applied for the assays.

Thymol, and the essential oils from *Menta* spp. and *Rosmarinus officinalis*, have been used against *E*. *granulosus* cells derived from the germinal layer of cysts, resulting in a loss of viability of 63% at 5 μg·mL^-1^ for thymol and between 71% and 82% for the other extracts at 10 μg·mL^-1^, after seven days of exposure, similar to the effects caused by ABZ at the same concentration and exposure time [193]. Interestingly, recent investigations have indicated that germinative (stem) cells might be less sensitive to chemotherapy because they express a beta-tubulin isoform with limited affinity to benzimidazoles [181]. Thus, although preliminary interesting, the results obtained by Albani et al. [193] should be further investigated regarding the effect of ABZ or any other compound against the different cell types present in a preparation of cells from the germinal layer of hydatid cysts.

Some activity against *E*. *granulosus* cysts *in vitro* and *in vivo* was detected when *Origanum vulgare* essential oil was used [175,194]. Similarly, extracts of *Zataria multiflora* showed activity against *E*. *granulosus* in the murine model, when given orally at infection (100% activity) or at 8 months post-infection (93% activity) [88,89], showing that plant extracts containing thymol could be useful for the treatment of CE patients.

Menthol (S1 Fig, compound 50) and menthol-rich extracts have been tested *in vitro* against *E*. *granulosus* protoscoleces and cysts, and also against germinal layer cells (see above), with variable results (Table 4). As for thymol, menthol as pure compound showed high (100%) activity against protoscoleces [190], but the essential oil obtained from *Mentha* spp. evidenced sub-optimal efficacy against both protoscoleces and cysts [194,195]. Differences in activity are most probably related with the final concentration of the active compound in extracts, reaching levels that could be not enough to exert the maximal activity.

#### Plant and fungus extracts

Eight additional plant extracts and three fungi extracts have been described in the literature as potential scolicidal agents (Table 4). Plants and fungi from which extracts were obtained were: *Allium sativum* [196–198], *Corylus* spp. and *Curcurbia* spp. [196], *Mallotus philippinensis* [199], *Sambucus ebulus* [200], *Trametes robiniophila* murr [101], *Berberis vulgaris* [201], *Pestalotiopsis* spp. [202], *Satureja khuzestanica* and *Olea europaea* [203], *Salvadora persica* [204], *Zingiber officinale* [205,206], *Curcuma longa* [206], *Nigella sativa* [207,208], *Pistacia atlantica* [209], *Cnidium monnieri* [210], *Punica granatum* [211], and *Penicillium* [212–214]. All of them showed ≥90% scolicidal activity with the exception of the hydroalcoholic extracts of *Corylus* spp. and *Curcurbia* spp. [197]. Effective concentrations ranged from 4 to 200 mg·mL^-1^ applied for 5 min to 3 h (Table 4). The most efficient extract in terms of concentration (4 mg·mL^-1^) and exposure time (5 min) was the aqueous extract of *Berberis vulgaris* [201]. Its main component is berberine, an isoquinolone alkaloid with activity against several pathogens [215]. Interestingly, berberine has shown anti-neoplastic effects [216], and this could be exploited to test its activity against *E*. *multilocularis*. Additionally, *Punica grantum* extracts showed activity *in vivo* against *E*. *granulosus* [211] and *Cnidium monnieri* osthole owed activity *in vivo* against *E*. *multilocularis* [210] secondary infection in mice, showing levels of efficacy similar to those of ABZ at the same regime dose (Table 4).

Extracts of the fungi *Pestalotiopsis* spp. and *Penicillium* spp. showed also to be fully effective against *E*. *granulosus* protoscoleces *in vitro*, although at prolonged (≥30 min) incubation times [202,212–214], thus their usefulness as scolicidal agents in intra-cyst application in limited. The third extract derived from a fungus (*Trametes robiniophila*) showed little effectivity against protoscoleces. Remarkably, this extract had deleterious effects on *E*. *granulosus* cysts when given orally to mice at 1.5 months post-infection [101], pointing out its potential application to treat CE. The extracts of *T*. *robiniophila* have attracted attention in the last few years as an anti-neoplastic compound [215].

### Conclusions and future directions

Drugs assayed against CE and AE can be classified in scolicidal and/or cysticidal. Application of scolicidal compounds either intra-cyst or peri-interventionally is used to minimise the risk of recurrence in CE patients during surgical or percutaneous procedures. To be effective, scolicidal agents must deliver their effects in the shortest therapeutic time period without eliciting undesirable adverse events. In recent years several compounds have been proposed as candidate drugs to substitute the recommended 20% saline solution as scolicidal agent. From these, glucose 50%, cetrimide, and H_2_O_2_ have a rapid effect on protoscoleces, but should be further evaluated for potential cytotoxic effects before being safely recommended for human use. Biogenic selenium particles, thymol, and several plant extracts have also shown promising scolicidal properties, and are regarded as non-toxic compounds at the concentrations used to exert their chemotherapeutic activity. Nevertheless, the main drawback of most of those compounds lies in the highly variable procedures involved in their in-house preparation, an inherent feature that makes difficult the assessment of the results obtained in different studies. In this regard, thymol is a commercially available compound and as such could be easily used in standardized trials to prove its therapeutic performance, particularly in those clinical cases in which biliary communication of cysts could advise against intra-cyst injection of 20% saline or alcohol as scolicidal agents.

The efficacy of scolicidal compounds at the systemic level (usually peri-operatively) has been far lesser investigated. In this specific clinical setting, candidate drugs should ideally combine high solubility and intra-cyst bioavailability with elevated scolicidal efficiency, easy administration regimen (preferably at low dose), and absence of side effects. Monensin, praziquantel, imanitib and 2-methoxyestradiol may well comply with most of these requirements, but further *in vivo* assays are needed to clearly demonstrate the adjunctive activity of these drugs at different regimes and also to prove their safety of use.

The advantages of defining a good scolicidal agent rely in its use to avoid secondary CE in patients. Disadvantages of testing only the scolicidal activity of drugs is that a good scolicidal agent can show no activity against the metacestode, and that scolicidal drugs are of no use in AE patients. In spite of this, majority of drug testing studies against *Echinococcus* are still performed *in vitro* against protoscoleces.

Drugs directed against the larval stages of *E*. *granulosus* and *E*. *multilocularis* should also share the same characteristics already mentioned for systemic scolicidal compounds. Unfortunately, most of the drugs assayed to date against CE and AE cysts do not fulfil these criteria. Many other factors may influence the parasitocidal activity of a given drug treatment against CE and AE. Particularly relevant is the time during the dosing interval of the administered drug, for which there is currently no consensus, not even for the best known compounds used until now, the members of the BMZ family. Other variables that could affect the effectivity of the treatment include the number, size, location, developmental stage and condition of cysts. Most of these variables are usually not considered during the chemotherapeutic evaluation process of a given compound. An additional and important variable affecting the outcome of drug treatment in CE and AE is the time post-infection in which drugs are used. In this respect, it has been shown by several authors that the *in vivo* treatment of CE and AE is more effective when applied at early post-infection times regardless of the drug used. This fact may explain, at least partially, the failure or limited success of therapies in CE and AE patients frequently reported in the literature, where treatments usually starts at a late, chronic stage of the disease. Similarly, the route of administration seems to be crucial for some compounds to exert their activity, since some drugs demonstrate parasitocidal properties in intraperitoneal administration but show little or no effect in more convenient regimes, e.g., oral administration.

Both CE and AE are still very much neglected diseases for which the current drug of choice is ABZ. Accessibility to ABZ is impaired by limited distribution and elevated cost not only in socioeconomically disadvantages areas, but also in a number of developed countries. Additionally, this compound seems to exert a parasitostatic, rather than a parasitocidal, effect against both parasites and no alternative drug is available for patients with AE who experienced severe side effects and cannot be treated with ABZ (or MBZ). This also seems to be the case for the vast majority of alternative drugs assayed to date, since *in vivo* assays have evidenced changes in cyst weight, but reduction in cyst number are rarely observed. Could ABZ or other drug from those that have already shown parasitostatic effects be parasitocidal under specific conditions? In an attempt to improve the intra-cyst bioavailability of ABZ, different formulations have been assessed aiming to increase the solubility, absorption, and stability of the drug. These improvements have translated into enhanced drug effectivity and should be further explored, e.g. by using nanoparticles that lead to increased intra-cyst drug levels, or novel drug enantiomers with higher bioavailability and activity.

An additional strategy to overcome the problem of drugs’ solubility is represented by the salification of sulfynil-benzimidazoles with sodium hydroxide solution. The proposed two-step production of the enantiomers of RBZNa and TCBZSONa (i.e. enantioseparation of racemic sulfoxides by HPLC on chiral stationary phase and successive transformation of the benzimidazole scaffold into a sodium salt form) is simple and it seems to be suitable for implementation on a semi-industrial or industrial scale. The main benefit of using water-soluble RBZNa and TCBZSONa salts is the possibility to prepare novel anthelmintic formulations with higher levels of bioavailability, downscaling currently recommended human dosage, thus possibly decreasing side adverse events than those currently reported for RBZ, ABZ and TCBZ.

A second line of investigation that seems promising is the use of therapies based on the combination of two or more agents, since some components have shown to act synergistically, e.g. together with ABZ, against the parasite, and importantly some of them have shown activity specifically against the stem cells of the parasite. Development of synergistic combinations of drugs can overcome toxicity and other side effects associated with high doses and/or long time dosage of single drugs.

From those molecules assayed in *in vivo* models as an alternative for BMZ compounds, very few have reached clinical use. One example is nitazoxanide, which showed high activity in preliminary *in vitro* and *in vivo* studies, but when used in patients obtained results were discouraging. Clinical translation of drugs assayed *in vivo* has not been tackled systematically, and number of treated patients have been usually low and their clinical status too variable to extract robust evidence-based conclusions. Thus, there is still an urgent need for defining new compounds or improved formulations of those already assayed, and also for a careful design of clinical protocols that could lead to the draw of a broad international consensus on the use of a defined drug, or a combination of drugs, for the effective treatment of CE and AE both in complicated and non-complicated cases.

Interestingly, data on the genome of both *E*. *granulosus* and *E*. *multilocularis* have been recently released [30,31]. These data have shown that the parasites display several sets of family molecules related both with the activity of already known drugs (e.g., praziquantel) and also with the activity of potentially new drugs that could find their targets in specific parasite enzymes (e.g., protein kinases). Reasons for the reduced efficacy reported for some drugs against larval stages and potential new targets could be extracted from these genomic data.

An orphan drug is defined as a compound that has been developed specifically to treat a rare medical condition. It is easier to gain marketing approval for an orphan drug, and there may be other financial incentives to encourage the development of compounds which might otherwise lack a sufficient profit motive. Despite the fact that both CE and AE are classified as orphan diseases (http://www.orpha.net/consor/cgi-bin/index.php?lng=EN), none of the drugs tested so far against these diseases have been specifically developed against them, but have previously been licensed based on the activity demonstrated against other infectious and non-infectious conditions.

## Supporting information

S1 Fig Chemical structures of the main bezimidazole compounds, synthetic compounds, and natural compounds described in the present review. (TIF)
